# Stage-Specific Reconstruction of Genome-Wide Genetic and Epigenetic Regulatory Networks Reveals Mechanistic Insights into Asthma Progression

**DOI:** 10.3390/ijms27093708

**Published:** 2026-04-22

**Authors:** Cheng-Wei Li, Rui-En Wu, Bor-Sen Chen

**Affiliations:** 1 Department of Electrical Engineering, Chang Gung University, No. 259, Wenhua 1st Rd., Guishan Dist., Taoyuan City 33302, Taiwan; 2 Department of Electrical Engineering, National Tsing Hua University, 101, Section 2, Kuang-Fu Road, Hsinchu 300044, Taiwan

**Keywords:** asthma, airway hyperresponsiveness, airway inflammation, immune system, system identification method, epigenetic modification

## Abstract

Asthma is a chronic respiratory disease characterized by airway hyperresponsiveness, obstruction, and persistent inflammation, arising from complex interactions among genetic, epigenetic, immune, and environmental factors. To elucidate the stage-specific molecular mechanisms underlying asthma progression, we constructed candidate genome-wide genetic and epigenetic networks (GWGENs) of human cells through large-scale biological database mining. Using a system order detection scheme, false-positive interactions were pruned to identify real GWGENs corresponding to three clinical stages of asthma: quiet, exacerbation, and follow-up. Core GWGENs were subsequently extracted from each real network using the principal network projection (PNP) method to highlight dominant regulatory structures and pathogenic pathways. Based on the inferred core networks, key stage-specific biomarkers were identified and further explored as potential drug targets. Drug–target relationships were investigated by integrating gene expression perturbation profiles from the Connectivity Map (cMap), comprising microarray data for 14,207 genes across 1327 compounds. This network-guided analysis enabled the qualitative design of multi-molecule drug combinations tailored to each disease stage. Our results suggest that asthma onset is associated with reduced innate immunity, increased disease susceptibility, and impaired endothelial barrier recovery influenced by microenvironmental factors such as cigarette smoke and lipopolysaccharides, together with genetic and epigenetic alterations. During the exacerbation stage, enhanced differentiation of T cells toward the T helper 2 lineage contributes to airway inflammation and tissue injury. In the follow-up stage, T helper 1–mediated responses are linked to mucus hypersecretion, airway obstruction, and sustained inflammation. Collectively, these findings demonstrate that a systems-level, network-based framework can uncover stage-specific pathogenic mechanisms of asthma and provide hypothesis-generating insights for network-informed drug repurposing strategies.

## 1. Introduction

Asthma is a prevalent chronic respiratory disease and a major contributor to global morbidity and mortality. It is characterized by abnormal immune responses to environmental stimuli, including animal allergens, cigarette smoke, pollen, and airborne particulates. Although these stimuli normally activate protective immune mechanisms, excessive immune activation leads to airway inflammation, bronchoconstriction, edema, and mucus hypersecretion, ultimately resulting in airflow limitation and impaired respiration. Recurrent asthma exacerbations are associated with sleep disturbance, fatigue, reduced physical activity, and school or work absenteeism, substantially diminishing quality of life and imposing a significant socioeconomic burden [1].

Despite advances in clinical management, asthma remains a major public health challenge due to incomplete disease control, persistent symptoms, and high healthcare utilization. Both genetic predisposition and environmental exposure contribute to asthma susceptibility; however, genetic variation alone cannot adequately explain disease heritability, phenotypic heterogeneity, or stage-specific progression. Increasing evidence indicates that epigenetic mechanisms provide an additional regulatory layer by modulating chromatin accessibility and gene expression without altering the underlying DNA sequence [2,3]. These mechanisms are highly responsive to environmental cues such as pollution, cigarette smoke, and microbial products, and play essential roles in immune regulation, cellular differentiation, and disease progression. Consequently, interactions between epigenetic regulation and the microenvironment are central to asthma pathogenesis and therapeutic response [4].

MicroRNAs (miRNAs) are key epigenetic regulators involved in a broad range of biological processes, including immune signaling, inflammation, and airway remodeling [5]. They have emerged as potential disease biomarkers and therapeutic targets in asthma, where dysregulated miRNA-mediated control contributes to immune imbalance and chronic airway inflammation. In addition to miRNAs, long non-coding RNAs (lncRNAs) and post-translational epigenetic modifications further expand the complexity of regulatory interactions underlying asthma. Such multilayered regulation cannot be fully characterized using reductionist or single-factor approaches, highlighting the need for integrative, systems-level analyses.

Existing network-based approaches, including co-expression networks [6], information-theoretic models [7], and machine learning-based gene regulatory network (GRN) inference methods [8], have significantly advanced the understanding of gene regulatory relationships. Co-expression network analysis methods, such as Weighted Gene Co-expression Network Analysis (WGCNA), have been widely used to identify gene modules and hub genes based on correlation patterns, whereas information-theoretic approaches, such as Algorithm for the Reconstruction of Accurate Cellular Networks (ARACNE), infer regulatory interactions by estimating mutual information and removing indirect associations. More recently, machine learning-based methods, including random forest and deep learning approaches, have been increasingly applied to capture complex and nonlinear regulatory relationships.

Despite these advances, most existing approaches primarily focus on static network reconstruction and lack explicit modeling of system dynamics and parameter estimation. In addition, they often do not simultaneously integrate genetic and epigenetic regulatory layers, nor do they adequately account for stage-specific variations during disease progression. These limitations hinder a comprehensive understanding of the dynamic and multi-layered regulatory mechanisms underlying complex diseases such as asthma.

To overcome these limitations, a more integrative and dynamic network modeling framework is required. In particular, approaches that incorporate system-level parameter estimation, multi-layer regulatory interactions, and structure-aware network reduction may provide improved interpretability and biological relevance in elucidating disease progression.

Recent studies have further emphasized the importance of integrating multi-omics data and incorporating dynamic modeling to better capture context-dependent regulatory mechanisms and disease progression, highlighting the need for more interpretable and mechanistically grounded network frameworks.

To address this challenge, we adopted a systems biology framework to construct genome-wide genetic and epigenetic networks (GWGENs) by integrating large-scale biological interaction databases with genome-wide microarray data. This approach enables comprehensive modeling of regulatory interactions among proteins, genes, miRNAs, lncRNAs, epigenetic modifications, and microenvironmental factors. Candidate GWGENs were first assembled through large-scale database mining, followed by system identification and model order detection to eliminate false-positive interactions. Principal network projection (PNP) was then applied to extract core GWGENs that capture the dominant regulatory architecture at each disease stage. These core networks were subsequently mapped onto Kyoto Encyclopedia of Genes and Genomes (KEGG) pathways to facilitate biological interpretation [9].

To elucidate stage-specific pathogenic mechanisms, asthma progression was stratified into three clinical stages: quiet, exacerbation, and follow-up. In the quiet stage, environmental stressors such as cigarette smoke, lipopolysaccharides, and hypoxia, together with genetic and metabolic perturbations involving *TLR4*, *CYBB*, *MMP12*, *NR1D2*, *GRB10*, and *PDHB*, contribute to reduced innate immune activity (*IRF1*, *IRF5*), impaired endothelial barrier recovery (*RHOB*), and increased asthma susceptibility (*CYFIP2*, *HLA-G*). During the exacerbation stage, epithelial–mesenchymal transition driven by *ZEB1*, *GATA1*, and *HOXA5*, inflammatory injury mediated by *BCL3* and *KLF4*, and dysregulated T-cell differentiation involving *IL10* and *IL4* collectively promote airway hyperresponsiveness and obstruction. In the follow-up stage, persistent inflammation and airway remodeling associated with *TRAF4*, *IL15*, *CCL7*, *STAT1*, *NFKB2*, *MUC2*, *SPDEF*, and *SOCS3* contribute to mucus hypersecretion, airway narrowing, and chronic airflow limitation.

Based on these stage-resolved core networks and pathogenic mechanisms, key regulatory genes were identified as potential drug targets. By integrating gene expression perturbation profiles from the drug–target database the Connectivity Map (cMap) [10], we further explored network-informed, multi-molecule drug repurposing strategies. This systems-level framework provides mechanistic insights into asthma progression and establishes a rational foundation for hypothesis-driven identification of stage-specific therapeutic strategies.

## 2. Results

### 2.1. Identification of GWGENs, Core GWGENs, and Stage-Specific Core Pathways

**Figure 1:**
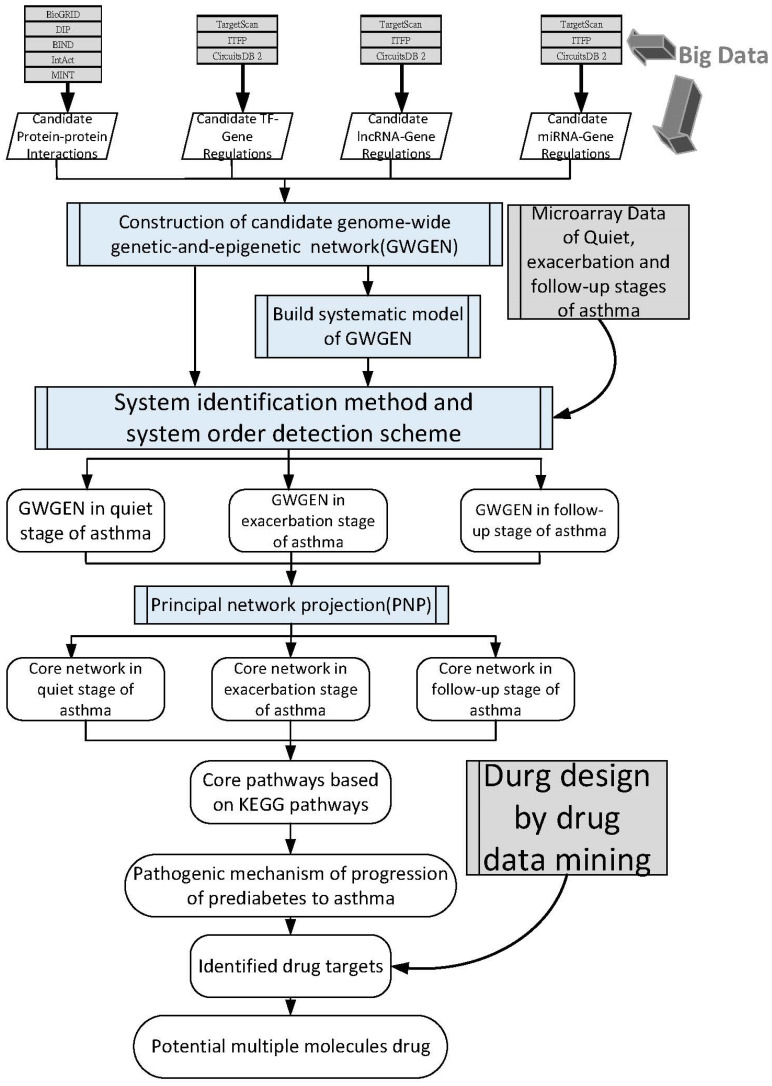
Overview of the systems biology workflow for constructing genome-wide genetic and epigenetic networks (GWGENs) to investigate stage-specific pathogenic mechanisms of asthma. Candidate GWGENs were constructed by large-scale database mining, integrating protein–protein interactions, transcription factor–gene regulations, microRNAs (miRNAs)–gene regulations, and long non-coding RNAs (lncRNAs)–gene regulations. Genome-wide microarray data from the quiet, exacerbation, and follow-up stages of asthma were used for system identification and system order detection to infer real GWGENs. Principal network projection (PNP) was then applied to extract core networks, which were mapped onto Kyoto Encyclopedia of Genes and Genomes pathways to elucidate stage-specific pathogenic mechanisms and identify potential drug targets. Gray blocks indicate input data sources, blue blocks represent systems biology–based analytical procedures, and white blocks denote the resulting inferred networks and biological insights. This workflow highlights the integration of data-driven system identification and network projection to enable stage-specific and mechanistically interpretable modeling of asthma progression.

To provide an overview of the proposed analytical framework, the overall workflow for constructing GWGENs and identifying stage-specific pathogenic mechanisms is illustrated in Figure 1. The workflow consists of four major steps: (i) construction of candidate GWGENs by integrating large-scale biological interaction databases, including protein–protein interactions, transcriptional regulations, and non-coding RNA regulations; (ii) system identification and model order detection using microarray data to infer real GWGENs and remove false-positive interactions; (iii) extraction of core subnetworks using PNP to identify dominant regulatory structures; and (iv) biological interpretation through KEGG pathway mapping and identification of potential drug targets and multi-molecule drug combinations. This framework enables systematic investigation of stage-specific regulatory mechanisms across the quiet, exacerbation, and follow-up stages of asthma.

**Figure 2:**
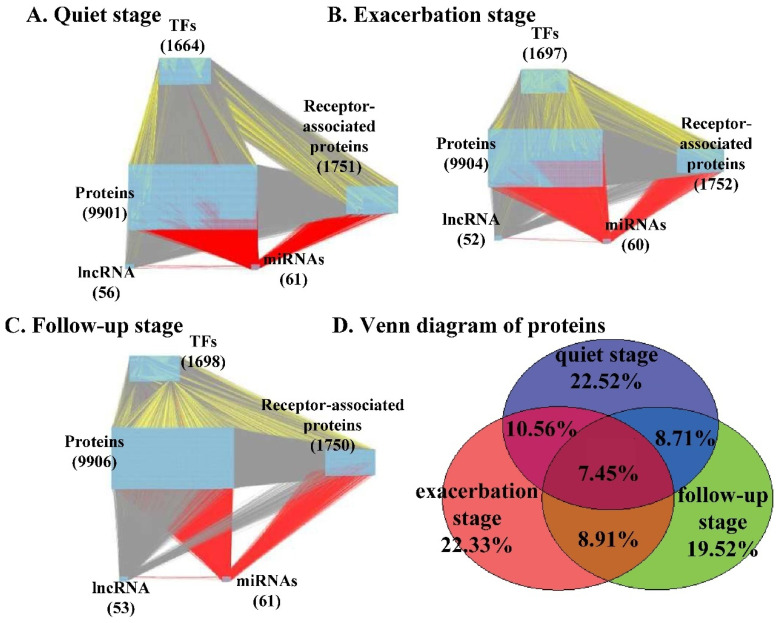
Real GWGENs inferred for different stages of asthma. (**A**) Quiet stage, (**B**) exacerbation stage, and (**C**) follow-up stage GWGENs after system identification and network refinement. Nodes represent transcription factors, proteins, lncRNAs, and miRNAs, and edges denote inferred regulatory interactions. (**D**) Venn diagram showing overlap and stage-specific distribution of proteins across the three real GWGENs. This comparison demonstrates that, despite similar network sizes, the underlying regulatory structures differ substantially across stages, reflecting stage-specific network reorganization rather than random variation. In panels (**A**–**C**), different colors represent distinct types of regulatory interactions and node categories, as defined in the figure legend.

The inferred genome-wide genetic and epigenetic networks for the quiet, exacerbation, and follow-up stages of asthma were reconstructed and visualized using Cytoscape version 3.10.4, as shown in Figure 2A–C. The quantitative characteristics of the inferred networks are summarized in Table 1 and Table 2. As shown in Table 1, although the candidate GWGEN contains 14,024 nodes, the number of nodes in the real GWGENs after system identification and AIC-based pruning is slightly reduced across the three stages (13,431–13,468 nodes). The distribution of node types—including transcription factors, lncRNAs, miRNAs, receptors, and proteins—remains largely consistent among stages, indicating that the core regulatory components are preserved despite network refinement. This suggests that the proposed framework effectively removes redundant or spurious nodes while maintaining the overall biological complexity of the system.

As shown in Table 2, a substantial reduction in the number of edges is observed after network refinement. The candidate GWGEN initially contains approximately 2.9 × 10^6^ interactions, which are pruned to approximately 7.3–8.0 × 10^5^ edges in the real GWGENs. Despite this significant reduction, the relative distribution of interaction types—including transcriptional regulation (T–G), protein–protein interactions (PPIs), and miRNA- or lncRNA-mediated regulation—remains comparable across the quiet, exacerbation, and follow-up stages. This indicates that the AIC-based system identification process effectively eliminates false-positive interactions while preserving essential regulatory structures and interaction diversity.

A Venn diagram summarizing the overlap of proteins across the three stages is provided in Figure 2D. Although the total number of proteins identified at each stage was comparable, approximately 22% of the proteins were unique to a specific stage, indicating substantial stage-specific network reorganization. Functional enrichment analysis of stage-specific networks was performed using DAVID, and the enriched cellular functions and pathways are summarized in Table 3.

Because the complete inferred networks were highly complex and not directly interpretable, we applied PNP to extract core subnetworks based on projection values defined in Equation (15). The resulting core GWGENs for the quiet, exacerbation, and follow-up stages are presented in Supplementary Figures S1–S3, respectively.

To investigate pathogenic mechanisms across disease stages, we selected the principal nodes ranked within the top 3000 projection values as core nodes and reconstructed their upstream and downstream regulatory relationships. In addition to canonical protein–protein interactions, epigenetic modifications—including ubiquitination/deubiquitination, acetylation/deacetylation, and phosphorylation/dephosphorylation—were incorporated into the network inference.

Epigenetic effects were inferred from the estimated basal activity terms in the stochastic protein–protein interaction network model, which capture regulatory influences beyond direct interactions. Proteins whose basal activity exceeded a predefined threshold were inferred to be subject to epigenetic modification. Annotation from NCBI gene descriptions was further used to support these inferences. For example, a protein exhibiting elevated basal activity and interacting with a protein annotated as a deubiquitination enzyme was inferred to undergo deubiquitination.

Similarly, in the gene regulatory network model, the basal regulatory term represents non-canonical genetic regulation. Genes with basal levels exceeding a predefined threshold were inferred to be influenced by DNA methylation or demethylation.

**Figure 3:**
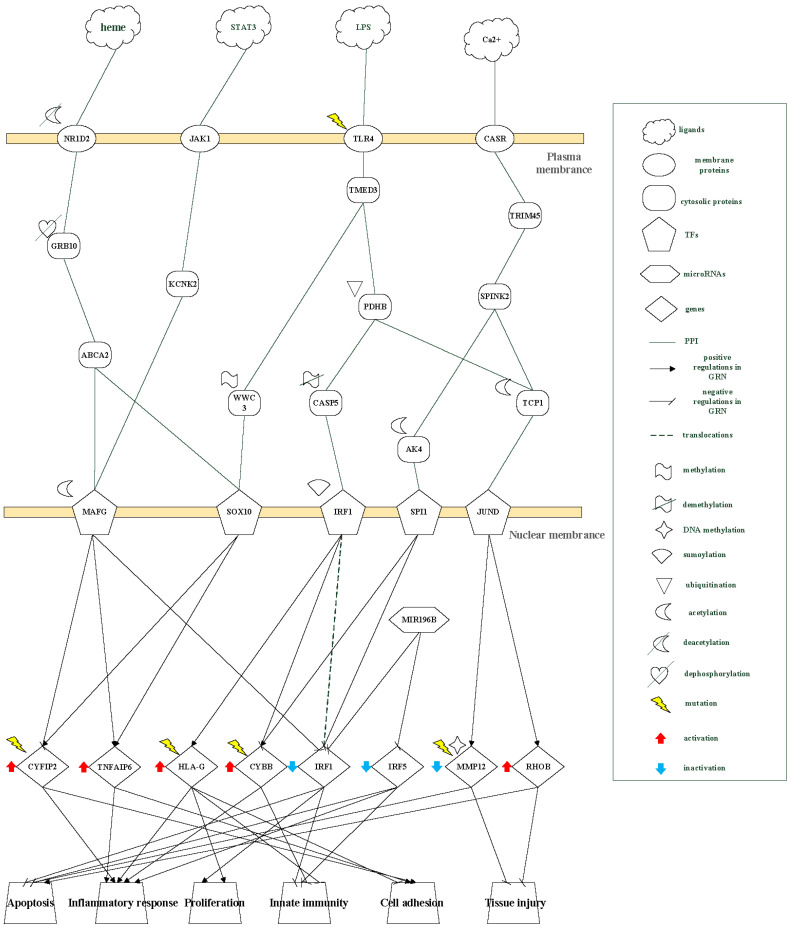
The pathogenic mechanism of core pathways in the quiet stage of asthma. At the quiet stage, the receptors NR1D2, JAK1, TLR4, and CASR transduce signals to transcription factors (TFs) MAFG, SOX10, IRF1, SPI1, and JUND through signaling transduction proteins. These regulatory interactions are associated with activation of target genes CYFIP2, TNFAIP6, HLA-G, and CYBB, and inhibition of IRF1, IRF5, and MMP12. The inferred regulatory patterns are associated with anti-apoptosis, inflammatory response, cell proliferation, reduced innate immunity, and tissue injury. This figure illustrates how multiple upstream signals converge to regulate key transcription factors and downstream targets, leading to impaired innate immunity, altered cell adhesion, and increased susceptibility to environmental stimuli in the quiet stage. These regulatory interactions are associated with immune modulation, cell adhesion, and early inflammatory responses.

**Figure 4:**
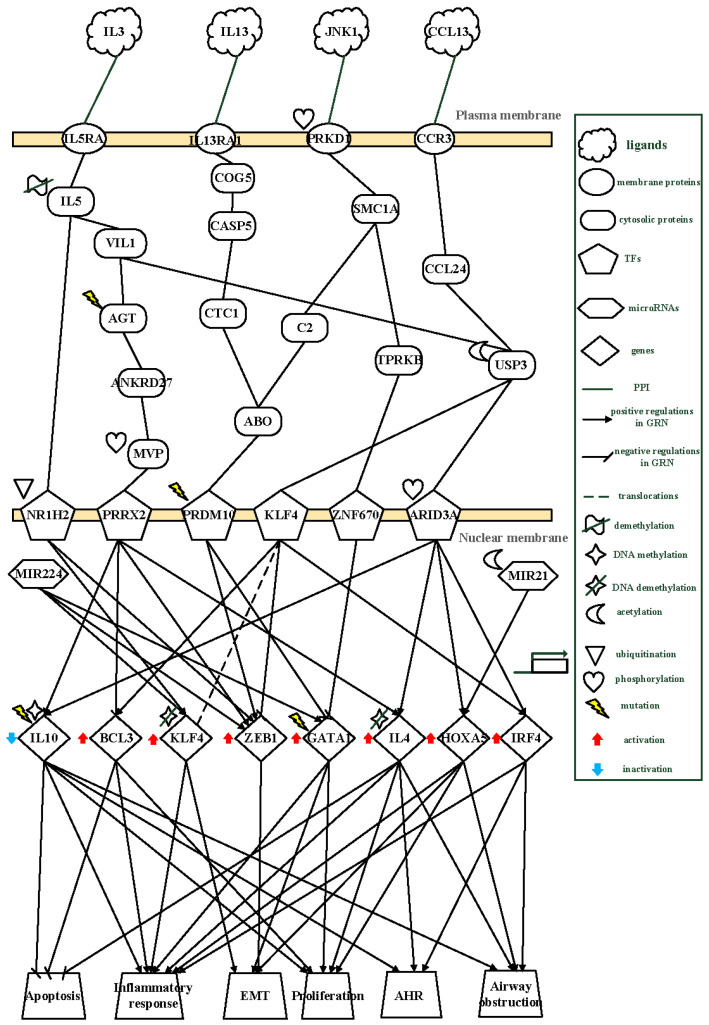
The pathogenic mechanism of core pathways in the exacerbation stage of asthma. At the exacerbation stage, the receptors IL5RA, IL13RA1, PRKD1, and CCR3 transduce signals to transcription factors (TFs) NR1H2, PRRX2, PRDM10, KLF4, ZNF670, and ARID3A through signaling transduction proteins. These regulatory interactions are associated with activation of target genes BCL3, KLF4, ZEB1, GATA1, IL4, HOXA5, and IRF4, and inhibition of IL10. The inferred regulatory patterns are associated with anti-apoptosis, inflammatory response, cell proliferation, EMT-related processes, AHR, and airway obstruction. This figure highlights coordinated Th2-associated signaling and regulatory interactions that are associated with epithelial remodeling and airway hyperresponsiveness during exacerbation.

**Figure 5:**
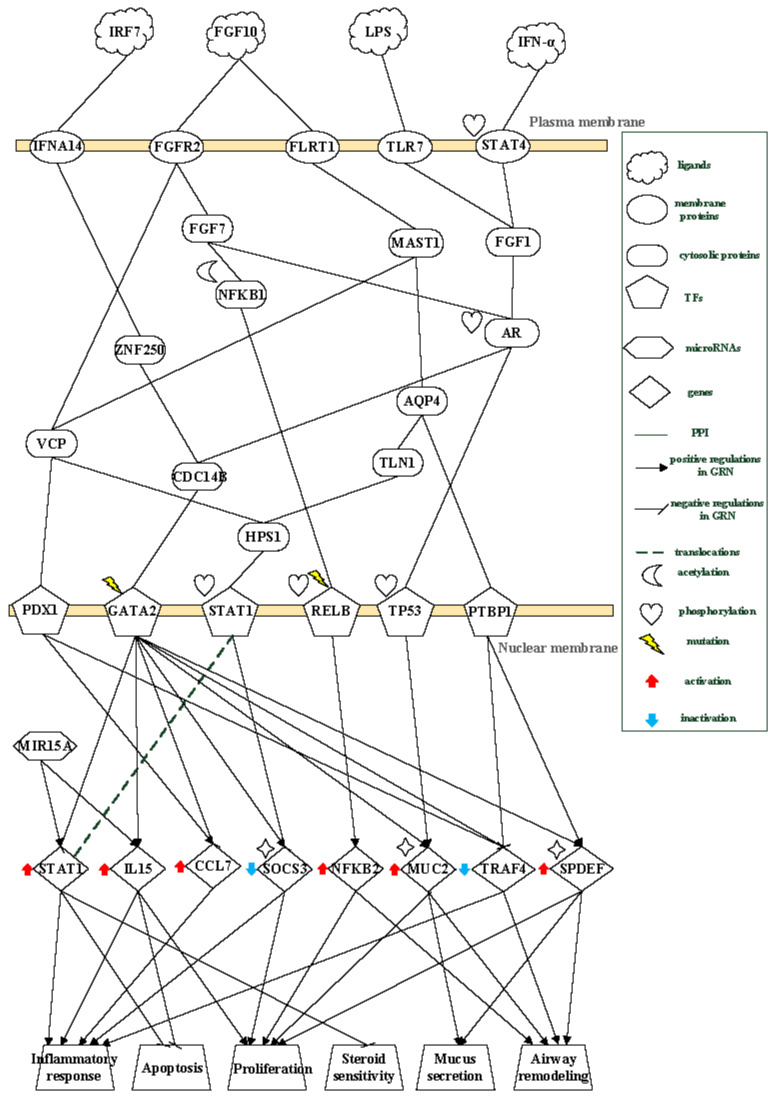
The pathogenic mechanism of core pathways in the follow-up stage of asthma. At the follow-up stage, the receptors IFNA14, FGFR2, FLRT1, STAT4, and TLR7 transduce signals to transcription factors (TFs) PDX1, GATA2, STAT1, RELB, TP53, and PTBP1 through signaling transduction proteins. These regulatory interactions are associated with activation of target genes STAT1, IL15, CCL7, NFKB2, MUC2, and SPDEF, and inhibition of SOCS3 and TRAF4. The inferred regulatory patterns are associated with anti-apoptosis, inflammatory response, cell proliferation, steroid responsiveness, mucus secretion, and airway remodeling. This figure illustrates regulatory interactions associated with persistent inflammation, mucus hypersecretion, and airway remodeling, reflecting progression toward chronic airway pathology and reduced therapeutic responsiveness.

To characterize disease progression from the quiet stage through exacerbation to the follow-up stage, the inferred core GWGENs were mapped onto KEGG pathways to extract corresponding core signaling pathways. The resulting stage-specific core pathways for the quiet, exacerbation, and follow-up stages are shown in Figure 3, Figure 4 and Figure 5, respectively. It should be noted that the transcriptomic data used in this study were derived from peripheral blood samples. Therefore, the inferred regulatory mechanisms represent systemic immune-associated regulatory signatures related to asthma progression, rather than direct measurements of airway tissue-specific processes. The identified pathways are interpreted as network-level associations that may reflect underlying disease mechanisms.

### 2.2. Core Pathways of Quiet Stage Asthma

As illustrated in Figure 3, the transcription factor MAFG is regulated by two major signaling routes in the quiet stage, both converging on acetylation-dependent modulation. In the first route, heme-mediated oxygenase–carbon monoxide signaling activates NR1D2, leading to deacetylation of the receptor and subsequent dephosphorylation of GRB10. Signal propagation through ABCA2 then results in activation of MAFG. In the second route, STAT3-associated growth hormone receptor signaling is transmitted via KCNK2 and JAK1, which also leads to activation of MAFG.

Activated MAFG promotes expression of *TNFAIP6*, which is involved in inflammatory responses and cell adhesion, while repressing *IRF1*, thereby contributing to inflammatory activity and reduced innate immune function. These regulatory effects are consistent with functional enrichment results, which highlight pathways associated with cytoskeletal regulation, cell adhesion, innate immunity, apoptosis, and inflammatory signaling in the quiet stage.

Specifically, enrichment of actin cytoskeleton regulation involving *CYFIP2* is associated with asthma susceptibility, while cell adhesion molecules such as *HLA-G* further contribute to immune modulation. Toll-like receptor–related signaling involving *IRF5* supports innate immune responses, whereas MAPK-associated signaling mediated by JUND is linked to *MMP12*-related apoptotic processes. In addition, pathways associated with cancer-related signaling include SPI-mediated regulation of *CYBB*, which contributes to inflammatory responses.

Analysis of the PNP-derived core networks indicates that multiple upstream receptors and transcription factors converge on five key genes—*CYFIP2*, *HLA-G*, *IRF1*, *IRF5*, and *RHOB*—each playing a distinct role in quiet-stage pathogenesis. *CYFIP2* and *HLA-G* are primarily involved in inflammatory signaling and immune evasion, *IRF1* and *IRF5* regulate innate immune activity and airway remodeling, and *RHOB* contributes to tissue repair. All five genes exhibit sensitivity to epigenetic regulation, including DNA methylation and acetylation, as well as environmental perturbations, supporting their identification as stage-specific and potentially druggable biomarkers in the quiet stage.

Collectively, these findings suggest that the quiet stage is characterized by a dysregulated yet not fully amplified inflammatory state at the systemic regulatory level. The inferred regulatory network reveals impaired innate immune activity, as indicated by suppression of *IRF1* and *IRF5*, together with enhanced expression of *TNFAIP6* and *HLA-G*, which are associated with inflammatory modulation and cell adhesion. In parallel, the coexistence of *MMP12*-mediated tissue injury and *RHOB*-associated repair processes suggests an imbalanced regulation between damage and repair.

These regulatory features may be associated with compromised epithelial barrier integrity and promote abnormal cell–cell interactions, thereby increasing susceptibility to environmental stimuli. Rather than triggering acute inflammation, such mechanisms appear to prime the airway microenvironment for subsequent exacerbation by establishing a vulnerable and functionally imbalanced immune state.

### 2.3. Core Pathways of the Exacerbation Stage of Asthma

As illustrated in Figure 4, a core regulatory pathway in the exacerbation stage involves ubiquitination-dependent inactivation of the transcription factor NR1H2. In this pathway, the cytokine IL-3 initiates signaling through its receptor *IL5RA*, and the inferred regulatory pathway is associated with demethylation-related epigenetic regulation linked to *IL5* expression. Elevated *IL5* expression is characteristic of asthma exacerbation and Th2 polarization and is associated with subsequent ubiquitination and functional suppression of NR1H2. Loss of NR1H2 activity results in increased expression of the downstream transcription factors KLF4 and ZEB1, thereby promoting inflammatory signaling and epithelial–mesenchymal transition (EMT).

Functional enrichment analysis based on KEGG pathways highlights several biological processes relevant to disease exacerbation. Cytokine–cytokine receptor interactions involving *IL5* are linked to inflammation and airway hyperresponsiveness, while chemokine signaling mediated by *CCR3* and *CCL24* contributes to inflammatory cell recruitment. In addition, regulation of cytokine production involving *IL10* modulates inflammatory responses, and hematopoietic cell lineage pathways involving *IL4* further amplify immune activation.

To identify therapeutic targets specific to the exacerbation stage, candidate biomarkers were prioritized according to their centrality within the core GWGEN and their mechanistic involvement in key pathological processes. Pathogenic hubs including KLF4, ZEB1, *IL4*, and *HOXA5* drive inflammation, EMT, aberrant cellular proliferation, airway hyperresponsiveness, and luminal obstruction. In contrast, *IL10* serves as a protective regulatory node whose restoration may re-establish anti-inflammatory control. All five genes exhibit epigenetic regulation and environmental responsiveness and are amenable to pharmacological intervention. Consequently, KLF4, ZEB1, *IL4*, *HOXA5*, and *IL10* were selected as stage-specific, druggable biomarkers for asthma exacerbation.

These results indicate that the exacerbation stage is dominated by a coordinated shift toward Th2-driven inflammation and epithelial remodeling. Mechanistically, *IL3*–IL5RA-mediated signaling induces demethylation of *IL5* and ubiquitination-dependent inactivation of *NR1H2,* leading to upregulation of KLF4 and ZEB1, which are associated with EMT-related processes. In parallel, IL13-associated signaling further amplifies EMT through PRDM10-mediated activation of ZEB1, while the CCL13–CCR3 axis promotes *CCL24* expression and subsequent activation of KLF4 and BCL3, contributing to eosinophil proliferation and sustained inflammatory responses.

These interconnected regulatory pathways establish a mechanistic link between immune polarization and structural remodeling, forming a positive feedback loop that reinforces both inflammation and EMT. This coupling of immune activation and epithelial transformation may contribute to the deterioration of airway function, including increased airway hyperresponsiveness and airway obstruction observed during exacerbation.

### 2.4. Core Pathways of the Follow-Up Stage of Asthma

As illustrated in Figure 5, activation of the transcription factor PDX1 in the follow-up stage is mediated by two FGF10-dependent signaling routes. In one route, FGF10 engages FGFR2 and transduces signals through VCP, resulting in activation of PDX1. In a parallel route, FGF10 signals via FLRT1 and MAST1, and PDX1 activation occurs when VCP integrates the MAST1-mediated signal. Activated PDX1 subsequently induces expression of *CCL7*, which contributes to inflammatory responses mediated by immune cell recruitment.

Functional enrichment analysis based on KEGG pathways highlights several processes associated with late-stage asthma pathology. JAK–STAT signaling involving STAT1 is linked to persistent inflammatory responses, while insulin signaling mediated by *SOCS3* modulates obesity-related metabolic processes. Neurotrophin-associated signaling involving RELB contributes to airway remodeling through regulation of NFKB2, and apoptosis-related pathways involving TRAF4 influence cellular proliferation.

Within the follow-up stage, several pathogenic hubs collectively drive disease persistence and structural airway changes. NFKB2 promotes airway smooth muscle proliferation and remodeling, whereas *MUC2* and *SPDEF* coordinate mucus hypersecretion and luminal obstruction. STAT1 contributes to sustained inflammation and reduced steroid responsiveness. In contrast, *SOCS3* functions as a critical negative regulator of inflammation and represents a potential target for functional restoration. Based on their central roles in the core GWGEN and mechanistic relevance, *NFKB2*, *MUC2*, *SPDEF*, *STAT1*, and *SOCS3* were identified as stage-specific, druggable biomarkers in the follow-up phase of asthma.

Taken together, these findings suggest that the follow-up stage represents a transition toward chronic airway remodeling and persistent inflammation. Mechanistically, FGF10–FGFR2–mediated signaling, together with downstream activation of VCP and PDX1, is associated with sustained epithelial remodeling and inflammatory gene expression, including *CCL7*. In parallel, interferon-related signaling, particularly through IFNA14–STAT1/STAT4 pathways, reflects ongoing immune dysregulation and contributes to reduced steroid responsiveness.

The coordinated regulation of *NFKB2*, *MUC2*, and *SPDEF* indicates persistent activation of mucus production and epithelial differentiation, which are key features of airway obstruction. In addition, the AQP4–HPS1–STAT1 axis promotes inflammatory amplification through suppression of *SOCS3*, a negative regulator of cytokine signaling. Although *SOCS3* may still exert a compensatory anti-inflammatory effect, its epigenetic repression limits its regulatory capacity.

This imbalance between sustained pro-inflammatory and remodeling signals and insufficient compensatory regulation likely drives long-term structural changes, including fibrosis, airway wall thickening, and luminal narrowing. Collectively, these mechanisms contribute to disease chronicity and increased susceptibility to environmental stimuli.

## 3. Discussion

### 3.1. The Main Pathogenic Progression Mechanism in the Quiet Stage

We constructed core pathways to investigate genetic and epigenetic mechanisms during the quiet stage using a systems biology framework combined with PNP-based analyses. As shown in Figure 3, NR1D2 is activated by heme and participates in the regulation of metabolic and inflammatory processes. Exposure to cigarette smoke [11,12] can induce deacetylation of NR1D2, which in turn leads to dephosphorylation of GRB10 [13] and disruption of immune balance, manifested as reduced innate immunity [14]. Inactivated GRB10 subsequently promotes activation of MAFG through ABCA2-mediated signaling.

In parallel, STAT3 signaling associated with growth hormone receptor activity binds to JAK1 and transduces signals through KCNK2, resulting in activation of MAFG. Acetylated MAFG positively regulates *TNFAIP6*, which is involved in inflammatory responses [3,15,16] and cell adhesion, while negatively regulating *IRF1*, thereby contributing to reduced innate immunity. *TNFAIP6* plays a role in cell–cell and cell–matrix adhesion as well as eosinophil migration, whereas nuclear translocation of *IRF1* supports macrophage-mediated innate immune responses [17] and airway epithelial homeostasis [18]. These regulatory patterns are consistent with metabolic dysregulation, including hypoglycemia and insulin signaling imbalance associated with reduced GRB10 [19], as well as obesity-related metabolic disturbances linked to reduced NR1D2 activity.

Lipopolysaccharide (LPS) binding to TLR4 initiates downstream signaling through TMED3, and genetic susceptibility, such as variation in TLR4, may exacerbate this response [20]. TMED3-associated signaling increases methylation of WWC3, a modification that has been linked to cigarette smoke exposure [21,22]. Increased WWC3 methylation leads to activation of SOX10, which subsequently induces *CYFIP2*, thereby promoting asthma susceptibility through enhanced T-cell adhesion and inflammatory differentiation [23]. In addition, WWC3 methylation has been associated with proliferative cellular responses.

**Figure 6:**
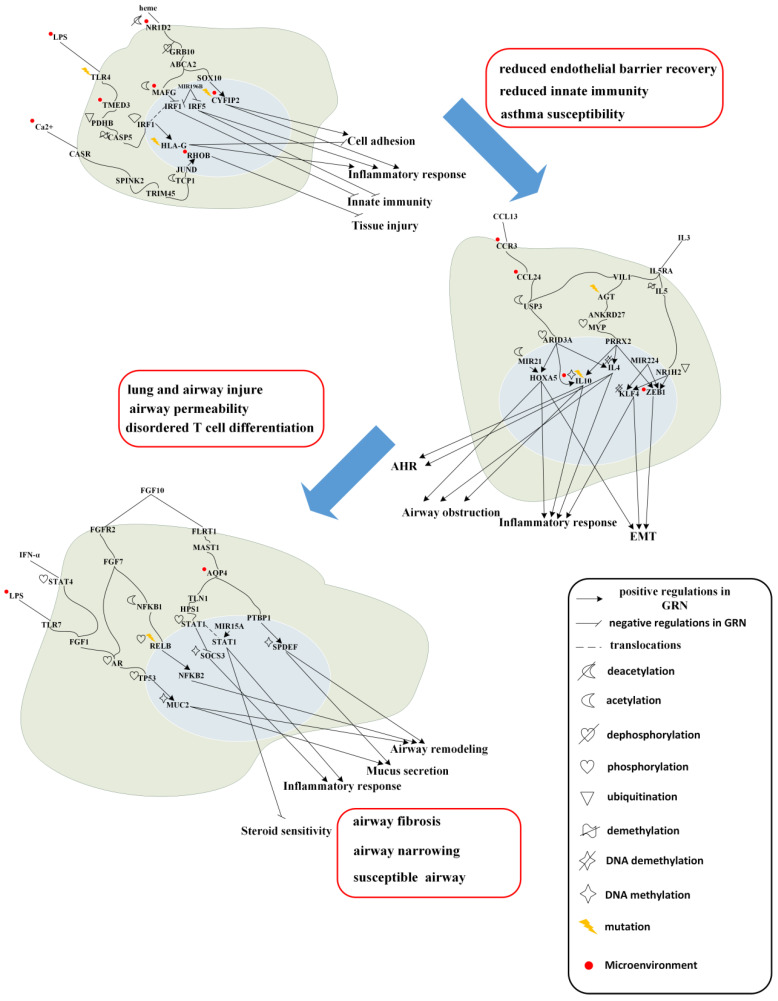
The overall pathogenic progression mechanism of asthma. The overall pathogenic progression mechanism is summarized by the important symptom factors of each stage from Figure 3, Figure 4 and Figure 5 including the mutations, microenvironments, epigenetic modifications and so on. The symptom factors could cause substantial change for the patient, such as reduced endothelial barrier recovery, reduced innate immunity and asthma susceptibility at the quiet stage; lung and airway injury, airway permeability, and disordered T cell differentiation at the exacerbation stage; airway fibrosis, airway narrowing and airway susceptibility at the follow-up stage. This integrated view highlights the progressive transition from immune dysregulation to inflammatory amplification and ultimately to chronic airway remodeling, providing a systems-level interpretation of asthma progression.

Calcium signaling also contributes to quiet-stage regulation. Activation of calcium-sensing receptor (CASR) signaling through TRIM45/SPINK2 and TCP1 results in activation of JUND, which upregulates *MMP12* and *RHOB*. *MMP12* is associated with lung injury and apoptosis, whereas *RHOB* contributes to tissue repair. Cigarette smoke exposure and dysregulated calcium signaling can reduce DNA methylation of *MMP12* [22,24], thereby enhancing its expression, while TCP1 is involved in epithelial remodeling and repair processes [25]. These quiet-stage regulatory mechanisms are summarized in Figure 6.

Our network-based workflow (Figure 1), which integrates a comprehensive candidate interactome, AIC-driven pruning to infer real genome-wide genetic and epigenetic networks, and PNP to extract core subnetworks, revealed multi-step regulatory cascades that are difficult to capture using conventional analysis approaches. These cascades follow a hierarchical organization from receptors through epigenetic modifiers and transcription factors to downstream effectors.

To further evaluate the robustness of the inferred networks, we note that the resulting regulatory structures are highly unlikely to arise from random connectivity. Specifically, the candidate GWGEN contains 14,024 nodes, corresponding to nearly 2 × 10^8^ possible pairwise interactions. After AIC-based pruning and system identification, only approximately 8 × 10^5^ edges are retained in the real GWGENs across different stages. The probability of obtaining such a structured yet sparse network from the full combinatorial space by random selection is therefore extremely low.

Moreover, as shown in Figure 2D, although the total number of proteins in the inferred networks is comparable across the quiet, exacerbation, and follow-up stages, only a small fraction of proteins are shared among stages. This limited overlap suggests that the inferred networks capture stage-specific regulatory reorganization rather than reflecting a common or random network structure.

These observations support the robustness and biological relevance of the proposed framework and indicate that the identified regulatory interactions are not artifacts of network size or density, but instead reflect condition-specific molecular mechanisms underlying asthma progression.

Representative examples include smoke-induced deacetylation of NR1D2, which leads to dephosphorylation of GRB10 and subsequent activation of MAFG; an LPS–TLR4–TMED3–associated methylation switch that induces SOX10 and subsequently activates *CYFIP2*; and a calcium-dependent signaling axis involving CASR, TCP1, and JUND, which balances protective *RHOB* signaling against injurious *MMP12* activity. These findings demonstrate the ability of the proposed framework to uncover stage-specific regulatory structures at the network level.

### 3.2. The Pathogenic Progression Mechanism of Exacerbation Stage of Asthma

As shown in Figure 4, IL-3, a cytokine/chemokine signal, promotes differentiation of T cells into T helper type 2 (Th2) cells. IL-3 is produced by T cells [26] and, through binding to IL5RA, induces demethylation of *IL5*. Elevated *IL5* expression is indicative of asthma exacerbation and Th2 differentiation and leads to ubiquitination-mediated inactivation of the transcription factor NR1H2 [27]. NR1H2 normally participates in cholesterol metabolism and anti-inflammatory responses [28]; its inactivation results in upregulation of the target genes *KLF4* and *ZEB1*.

*ZEB1* is activated through positive regulation by *MIR224*, NR1H2, PRRX2 [29], and PRDM10, and negative regulation by KLF4, thereby promoting EMT via reduced E-cadherin expression and disruption of epithelial tight junctions. In addition, *ZEB1* expression is increased by nickel (Ni) exposure in the microenvironment, which has been implicated in several lung diseases [30]. Nuclear translocation of KLF4 is promoted by NR1H2, *MIR224*, and DNA demethylation [31], inducing Th2 differentiation and inflammatory responses and further enhancing EMT through inhibition of E-cadherin [32,33]. In this pathway, obesity-related phenotypes may arise from metabolic dysregulation associated with reduced NR1H2 activity.

IL-13, secreted by Th2 cells and exacerbated by cigarette smoke exposure, binds to IL13RA1 and transduces signals to CTC1 through COG5 and CASP5. CTC1 promotes fibroblast proliferation downstream of EMT and relays signaling to PRDM10 via ABO, resulting in activation of mutant PRDM10, which is associated with IgE-mediated allergic responses driven by Th2 immunity [34,35]. Activated PRDM10 further upregulates *ZEB1*, thereby amplifying EMT through suppression of E-cadherin.

CCL13 engages CCR3 to induce *CCL24*, which is expressed on eosinophils and Th2 cells [36,37]. *CCL24* expression is enhanced by lipopolysaccharide (LPS) and promotes Th2 differentiation and eosinophilia, driving inflammatory responses [38]. *CCL24* subsequently acetylates USP3, leading to activation of KLF4, which upregulates *BCL3*. *BCL3* is positively regulated by KLF4 and negatively regulated by PRRX2, thereby promoting eosinophil proliferation and inhibiting T-cell apoptosis [34,39], resulting in sustained inflammation.

During asthma exacerbation, patients experience airway and lung inflammation, airway obstruction, and airway hyperresponsiveness (AHR). EMT-mediated epithelial barrier disruption, inflammation-driven tissue injury, and disordered T-cell differentiation collectively drive disease progression toward the follow-up stage. These exacerbation-stage mechanisms are summarized in Figure 6.

Our network-based approach identified multiple regulatory axes in the exacerbation stage that are difficult to detect using conventional analysis methods. First, IL-3–IL5RA signaling induces demethylation of the *IL5* locus and ubiquitination-mediated inactivation of NR1H2, leading to upregulation of *KLF4* and *ZEB1* and promoting Th2 differentiation and EMT. Second, IL-13–IL13RA1–CTC1 signaling activates mutant PRDM10 via ABO, further amplifying *ZEB1*-mediated EMT and IgE-associated allergic responses. Third, CCL13–CCR3 engagement induces *CCL24* expression, triggering USP3 acetylation and KLF4 activation and culminating in *BCL3*-mediated eosinophil proliferation and T-cell survival. These layered epigenetic and transcriptional circuits emerged only after construction of a comprehensive candidate network, pruning by AIC, and extraction of the core subnetwork via PNP.

### 3.3. The Pathogenic Progression Mechanism of Follow-Up Stage of Asthma

In the core pathways of the follow-up stage of asthma, fibroblast growth factor signaling plays a central role in airway remodeling. FGF10 binds to FGFR2 and initiates downstream signaling through VCP, a protein involved in lipid metabolism and cell growth, whose expression can be enhanced by cigarette smoke exposure [40]. Activation of VCP promotes the transcription factor PDX1, which subsequently upregulates the gene *CCL7*. Expression of *CCL7* contributes to inflammatory responses mediated by macrophages and neutrophils [41] and is positively regulated by PDX1 while being negatively regulated by GATA2.

Increased airway permeability and tissue injury enhance interferon-related signaling. IRF7 engages IFNA14 and activates GATA2 through the signaling intermediates ZNF250 and CDC14B. Activated GATA2 contributes to lung inflammation [42], which is associated with upregulation of *IL15* and downregulation of *TRAF4*. The gene *IL15* promotes differentiation of T cells into T helper type 1 (Th1) cells, enhancing T-cell proliferation and survival [43], and is positively regulated by *MIR15A* while being negatively regulated by GATA2. In contrast, *TRAF4* normally suppresses inflammatory signaling by inhibiting NFKB2 [44]; however, its repression by PDX1, GATA2, and PTBP1 counteracts this protective effect.

FGF10–FGFR2 signaling also induces FGF7, which stimulates epithelial cell proliferation associated with airway remodeling [45]. FGF7 activates NFKB1 through acetylation, and activated NFKB1 phosphorylates RELB [46]. RELB subsequently upregulates *NFKB2*, a gene that promotes airway smooth muscle proliferation and contributes to airway remodeling. Mutational activation of RELB further enhances *NFKB2* expression [47], reinforcing remodeling processes.

In parallel, FGF10 signaling through FLRT1 and MAST1 activates AQP4, whose expression can be increased by lipopolysaccharide exposure [48]. AQP4 transduces inflammatory signals to HPS1 via TLN1. HPS1 contributes to pulmonary fibrosis and activates STAT1 [49]. Phosphorylated STAT1 suppresses *SOCS3*, a gene that normally inhibits T-cell proliferation and Th1 differentiation [50]. Epigenetic repression of *SOCS3* therefore amplifies inflammatory responses. AQP4 also activates PTBP1, leading to upregulation of *SPDEF*, which promotes goblet cell proliferation and mucus secretion, contributing to airway remodeling.

Interferon-α signaling through the JAK–STAT pathway further modulates immune dysregulation. IFN-α activates STAT4, which promotes Th1 differentiation under conditions of disordered T-cell regulation. Activated STAT4, together with TLR7 signaling in response to lipopolysaccharide, transduces signals through FGF1 to the androgen receptor (AR). Activated AR induces phosphorylation of TP53, which upregulates *MUC2*. Expression of *MUC2* drives mucus hypersecretion and goblet cell proliferation, leading to airway obstruction and remodeling [51]. In addition, nuclear translocation of STAT1 has been associated with steroid insensitivity [52], which may reduce therapeutic efficacy in chronic inflammation.

Collectively, during the follow-up stage, patients experience airway obstruction caused by excessive mucus secretion, persistent airway and lung inflammation driven by disordered T-cell differentiation, and progressive airway remodeling. These processes lead to fibrosis, increased airway wall thickness, and narrowing of the airway lumen, thereby heightening susceptibility to environmental stimuli.

### 3.4. Overall Progression for Drug-Target Identification and Multi-Molecule Drug Design at Each Stage

Mutations, epigenetic modifications, and microenvironmental perturbations collectively predispose individuals to asthma exacerbations. To design stage-specific multi-molecule drug combinations, key regulatory mechanisms were selected from the core pathways identified at each disease stage based on network projection values and evidence of epigenetic alteration or mutation.

Drug–target relationships were queried using the cMap database, which provides microarray expression profiles for a large collection of genes and compounds. Based on the inferred stage-specific regulatory mechanisms, candidate drug targets were identified and combined to restore dysregulated cellular functions within the core networks.

For the quiet stage, multiple compounds were selected to modulate targets involved in immune regulation, inflammation, and tissue repair. These compounds collectively act on CYFIP2, HLA-G, IRF1, IRF5, and RHOB, aiming to suppress inflammatory signaling while enhancing innate immune responses and cellular recovery.

For the exacerbation stage, candidate compounds were selected to suppress epithelial–mesenchymal transition, reduce inflammation, and alleviate airway obstruction. These compounds primarily target *IL4*, *KLF4*, *ZEB1*, *HOXA5*, and *IL10*, thereby modulating Th2-driven immune responses and limiting pathological remodeling.

For the follow-up stage, selected compounds were designed to reduce mucus hypersecretion, airway remodeling, and steroid resistance. These agents act on *NFKB2*, *MUC2*, *SPDEF*, *SOCS3*, and STAT1, targeting pathways associated with smooth muscle proliferation, goblet cell expansion, and chronic inflammation.

The final multi-molecule drug combinations were identified by integrating the effects of multiple compounds to collectively reverse the dysregulated gene expression patterns observed in each disease stage, as summarized in Table 4. This selection is based on a rule-based strategy that matches drug-induced gene expression changes from the cMAP database with disease-associated expression profiles.

**Figure 7:**
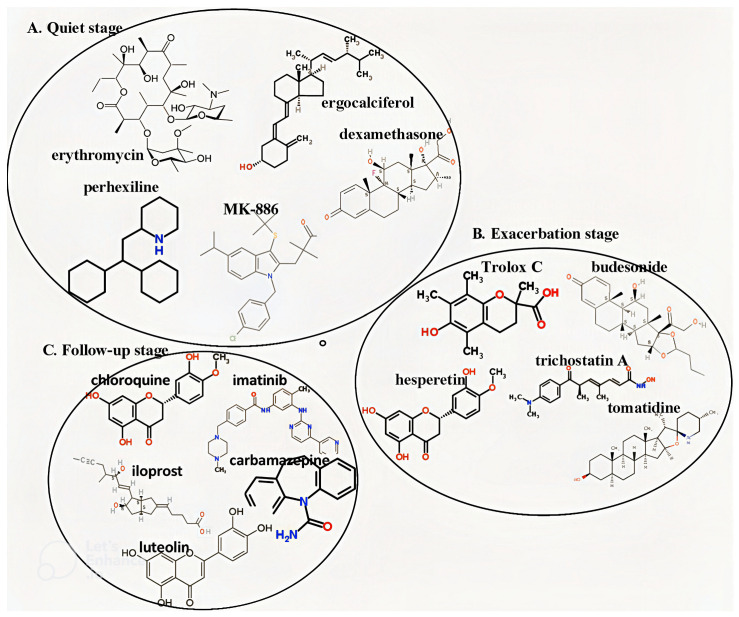
Two-dimensional structure of multiple-molecule drug for (**A**) quiet, (**B**) exacerbation and (**C**) follow-up stages. These compounds were selected based on their ability to target stage-specific regulatory mechanisms identified from the core GWGENs, supporting a network-informed strategy for multi-molecule drug design.

The two-dimensional chemical structures of the proposed multi-molecule drug combinations for each disease stage are illustrated in Figure 7. These combinations were derived from drug targets identified within the core pathways inferred from the real GWGENs, representing the final step of the proposed systems biology workflow.

It should be noted that the candidate compounds were selected from the cMAP database, which includes both clinically approved drugs and experimental small molecules. Therefore, not all identified compounds are currently used for asthma treatment. Nevertheless, the inclusion of clinically relevant drugs, such as corticosteroids (e.g., budesonide and dexamethasone), supports the biological validity of the proposed framework, while other compounds represent potential candidates for drug repurposing.

When evaluating candidate drugs for asthma therapy, compounds with established clinical safety profiles are more readily translatable. In contrast, some identified compounds may exhibit potential toxicity or off-target effects. For example, certain drugs included in the predicted combinations are known to have systemic toxicities and would require careful dose optimization and clinical monitoring. Therefore, the proposed multi-molecule drug combinations should be interpreted as computational predictions for hypothesis generation rather than directly applicable therapeutic regimens. Further experimental validation, pharmacokinetic analysis, and safety assessment are necessary to evaluate the feasibility and clinical applicability of these combinations.

## 4. Materials and Methods

### 4.1. Overview of the Construction of Real Genome-Wide Genetic and Epigenetic Networks

To investigate stage-specific pathogenic mechanisms of asthma, we first constructed candidate genome-wide genetic and epigenetic networks using large-scale database mining. Real networks were subsequently inferred by applying system identification to genome-wide high-throughput expression data. Core subnetworks were then extracted using the PNP method, enabling identification of representative miRNAs, lncRNAs, proteins, and genes or transcription factors from the inferred networks at each asthma stage, including the quiet, exacerbation, and follow-up stages. As summarized in Figure 1, the proposed framework integrates candidate network construction, system identification, and core network extraction to investigate stage-specific mechanisms of asthma.

### 4.2. Big Data Mining and Preprocessing of Asthma Transcriptomic Data

To characterize stage-specific pathogenic mechanisms of asthma, genome-wide microarray data were retrieved from the Gene Expression Omnibus (GEO) database (National Center for Biotechnology Information, Bethesda, MD, USA) of the National Center for Biotechnology Information (NCBI). A single dataset, GSE19301, was selected to minimize batch effects, as all samples were generated using the same oligonucleotide microarray platform under uniform experimental conditions.

The GSE19301 dataset comprises peripheral blood transcriptomic profiles from 118 adult asthma patients, yielding a total of 338 samples. These samples were collected across three clinically defined disease stages: the quiet stage (stable disease; 118 samples), the exacerbation stage (acute deterioration; 118 samples), and the follow-up stage (post-exacerbation recovery; 102 samples). Subjects with active infection, major intercurrent illness, allergen immunotherapy, pregnancy, or lactation were excluded to reduce potential confounding factors.

During the one-year study period, participants attended routine clinical visits every three months, during which asthma assessments and blood sample collection were performed. In addition to scheduled visits, subjects were instructed to attend the clinic as soon as possible at the onset of an exacerbation episode. A further assessment and blood sampling were conducted 14 days after symptom resolution to capture the follow-up stage. Accordingly, the three disease stages represent stable disease during routine monitoring, acute exacerbation within a 14-day symptomatic period, and recovery during the 14 days following an exacerbation.

Raw CEL files were downloaded from GEO and processed using a uniform preprocessing pipeline. Background correction and quantile normalization were performed using the robust multi-array average (RMA) algorithm implemented in the Bioconductor package. Probe sets were mapped to official NCBI gene symbols, and probes without unique gene annotations were removed. The resulting high-quality normalized expression matrix was subsequently used for downstream system identification and genome-wide network inference.

When multiple probe sets were mapped to the same gene symbol, the corresponding expression values were aggregated to obtain a single gene-level expression value. Specifically, the average expression value across all probes corresponding to the same gene was calculated. In addition, probe sets mapped to synonymous gene symbols were unified based on official NCBI gene annotations, and their expression values were also averaged to ensure consistency in gene representation. This preprocessing step resulted in a unique expression value for each gene used in downstream analysis.

### 4.3. Constructing Stochastic System Models of Protein–Protein Interaction Network (PPIN) and GRN in Candidate GWGENs for Stage-Specific Asthma

Candidate GWGENs were constructed to represent genome-wide genetic and epigenetic regulatory interactions underlying asthma progression at different disease stages. These networks integrated PPIs, transcription factor–gene regulations, lncRNA–gene regulations, and miRNA–gene regulations, as illustrated in Figure 1. To comprehensively cover candidate PPIs, curated human interaction data were integrated from multiple public databases, including BioGRID, the Database of Interacting Proteins (DIP), the Biomolecular Interaction Network Database (BIND), IntAct, and the Molecular Interaction Database (MINT). Regulatory interactions mediated by transcription factors, lncRNAs, and miRNAs were obtained from TargetScan, ITFP, and CircuitsDB 2. To ensure consistency across heterogeneous data sources, gene identifiers were standardized using both official and obsolete NCBI gene symbols.

Because candidate networks derived from large-scale database mining inevitably contain false-positive interactions, a modeling-based system identification framework was applied to infer real GWGENs for each asthma stage. Genome-wide microarray expression data corresponding to the quiet, exacerbation, and follow-up stages of asthma were incorporated to estimate regulatory parameters and prune spurious connections. System order detection based on the Akaike Information Criterion (AIC) was employed to substantially reduce the number of candidate interactions while preserving dominant and biologically relevant regulatory structures. Through this procedure, millions of initial candidate connections were reduced to stage-specific real GWGENs that capture core genetic and epigenetic regulatory mechanisms underlying asthma pathogenesis. The overall workflow of network construction and refinement is summarized in Figure 1, and the numbers of nodes and edges in the candidate and real GWGENs are reported in Table 1 and Table 2.

The PPIs in the candidate GWGENs were subsequently formulated using stochastic protein interaction equations, as described below.

(1) $$\begin{array}{l} p_{x}[n]=\sum_{\begin{array}{l} y=1\\ y\neq x \end{array}}^{Y_{x}}a_{xy}p_{x}\left[n\right]p_{y}\left[n\right]+b_{x,PPIs}+w_{x,PPIs}\left[n\right],\\ \mathrm{for}x=1,\;\ldots ,\;X,\;\mathrm{and}\;n=1,\;\ldots ,\;N \end{array}$$

where *X* represents the number of proteins and *N* represents the number of samples/patients; $p_{x}[n]$ represents the expression level of the *x*th protein; *a_xy_* represents the interaction ability between the *x*th protein and *y*th interactive protein; Y*_x_* indicates the total number of proteins interacting with the *x*th protein; *b_x_*_,*PPIs*_ denotes the basal level to model the epigenetic modification out of conventional protein interactions; $w_{x,PPIs}\left[n\right]$ represents the stochastic noise owing to the models of remainder and data fluctuation in the *x*th protein.

Because the gene expression level of GWGENs may be regulated by transcription factors (TFs)/proteins, miRNAs and lncRNAs. So, we need to construct a GRN of the candidate GWGEN.

The candidate GRN can be described as the following gene regulatory equations:

(2) $$\begin{array}{l} g_{i}[n]=\sum_{\begin{array}{l} o=1\\ o\neq i \end{array}}^{O_{i}}c_{io}p_{o}[n]+\sum_{\begin{array}{l} u=1 \end{array}}^{U_{i}}d_{iu}l_{u}[n]-\sum_{\begin{array}{l} r=1 \end{array}}^{R_{i}}e_{ir}g_{i}[n]k_{r}[n]+b_{i,GRN}+w_{i,GRN}[n],\\ \;\mathrm{for}\;i=1,\;\ldots ,\;I\;\mathrm{and}\;n=1,\;\ldots ,\;N \end{array}$$

where I represents the total number of genes and N denotes the number of samples; $g_{i}[n]$ represents the expression level of the *i*th gene; $p_{o}[n]$, $l_{u}[n]$ and $k_{r}[n]$ denote the expressions of the *o*th TFs, the *u*th lncRNA and the *r*th miRNA, respectively; $c_{io}$ denotes the transcription regulatory ability from the oth TF to the ith gene; $O_{i}$ indicates the total number of TFs binding to the *i*th gene; $d_{iu}$ is the transcription regulatory ability from the *u*th lncRNA to the *i*th gene; $U_{i}$ denotes the total number of lncRNAs binding to the ith gene; $e_{ir}$ ≥ 0 represents the post-transcriptional regulatory ability of the *r*th miRNA to inhibit the ith gene; $R_{i}$ is the total number of miRNAs inhibiting the *i*th gene; $b_{i,GRN}$ indicates the basal level of the *i*th gene expression; $w_{i,GRN}[n]$ represents the stochastic noise owing to the modeling residue and fluctuation of gene expression in the *i*th gene.

The expression level of lncRNA is affected by the regulation of TFs, proteins, lncRNAs and miRNAs. The lncRNA regulatory network (LRN) model of the candidate GWGENs can be described by regulatory equations as follows:

(3) $$\begin{array}{l} l_{h}[n]=\sum_{\begin{array}{l} o=1 \end{array}}^{O_{h}}S_{ho}p_{o}[n]+\sum_{u=1}^{U_{h}}\Gamma_{hu}l_{u}[n]-\sum_{r=1}^{R_{h}}\lambda_{hr}k_{r}[n]l_{h}[n]+\varphi_{h}+w_{h,LRN}[n]\\ \;\mathrm{for}\;h=1,\;\ldots ,\;H\;\mathrm{and}\;n=1,\;\ldots ,\;N \end{array}$$

where *H* is the total number of lncRNAs and n represents the number of samples; $l_{h}[n]$ represents the expression level of the *h*th lncRNA; $p_{o}[n]$, $l_{u}[n]$ and $k_{r}[n]$ represents the expression levels of regulatory TFs, lncRNAs and miRNAs, respectively; $S_{ho}$ denotes the transcription regulatory ability from the *o*th TF to the *h*th lncRNA; $O_{h}$ indicates the total number of TFs binding to the *h*th lncRNA; $\Gamma_{hu}$ is the transcription regulatory ability from the *u*th lncRNA to the *h*th lncRNA; $U_{h}$ denotes the total number of lncRNAs binding to the *h*th lncRNA; $\lambda_{rh}$ ≥ 0 denotes the post-transcriptional regulatory ability of the *r*th miRNA to inhibit the *h*th lncRNA; $R_{h}$ is the total number of miRNAs inhibiting the *h*th lncRNA; $\varphi_{h}$ indicates the basal level of the *h*th lncRNA expression; $w_{h,LRN}[n]$ represents the stochastic noise owing to the modeling residue and data fluctuation in the *h*th lncRNA.

The expression level of miRNA is affected by the regulation of TFs/proteins, lncRNAs and miRNAs. The candidate miRNA regulatory network (MRN) model of the candidate GWGENs can be described by regulatory equations as follows:

(4) $$\begin{array}{l} k_{z}[n]=\sum_{\begin{array}{l} o=1 \end{array}}^{Oz}\eta_{zo}p_{o}[n]+\sum_{u=1}^{U_{z}}\delta_{zu}l_{u}[n]-\sum_{r=1}^{R_{z}}\sigma_{zr}k_{r}[n]k_{z}[n]+\Phi_{z}+\zeta_{z}[n]\\ \;for\;z=1,\;\ldots ,\;Z\;\mathrm{and}\;n=1,\;\ldots ,\;N \end{array}$$

where *Z* is the total number of miRNAs and *N* represents the number of samples; $k_{z}[n]$ is the expression level of the *z*th miRNA; $p_{o}[n]$, $l_{u}[n]$ and $k_{r}[n]$ are the expression levels of regulatory TFs/proteins, lncRNAs and miRNAs; $\eta_{zo}$ denotes the transcription regulatory ability from the *o*th TF to the *z*th miRNA; $O_{z}$ indicates the total number of TFs binding to the *z*th miRNA; $\delta_{zu}$ is the transcription regulatory ability from the *u*th lncRNA to the *z*th miRNA; Uz denotes the total number of lncRNAs binding to the *z*th miRNA; $\sigma_{zr}$ ≥ 0 denotes the post-transcriptional regulatory ability of the *r*th miRNA to inhibit the *z*th miRNA; $R_{z}$ is the total number of miRNAs inhibiting the *z*th miRNA; $\Phi_{z}$ indicates the basal level of the *z*th miRNA expression; $\zeta_{z}[n]$ represents the stochastic noise owing to the modeling residue and data fluctuation in the *z*th miRNA.

The above methods and models are proposed to describe the genetic and epigenetic regulations and interactions of molecular mechanisms. In addition, the epigenetic modifications can contribute to both basal levels $b_{x,PPIN}$, $b_{i,GRN}$, $\varphi_{h}$ and $\Phi_{z}$ in Equations (1)–(4) because these basal levels are employed to model unknown interactions except those mentioned in these genetic interaction and regulation equations. When a basal level of PPIs exceeds a threshold, the protein with an overtaking threshold of basal level in the process of messaging was speculated that these core proteins may be affected by epigenetic modification such as acetylation, methylation, ubiquitination and phosphorylation. Moreover, the gene has an overtaking threshold of the basal level. In the process of messaging, it is speculated that this gene may be influenced by DNA methylation or DNA demethylation.

In this study, epigenetic and post-translational regulatory effects were not directly measured from the microarray data. Instead, they were inferred indirectly from the topology of the real GWGEN, especially from the PPI layer. Specifically, when a protein in the inferred network interacted with proteins functionally associated with methylation, demethylation, acetylation, deacetylation, phosphorylation, dephosphorylation, ubiquitination, or deubiquitination, the corresponding downstream regulatory pathway was interpreted as potentially involving the associated epigenetic or post-translational mechanism. Therefore, these inferred modifications should be regarded as network-based mechanistic associations rather than direct biochemical measurements of a specific DNA locus or protein modification event. The inferred epigenetic and post-translational mechanisms in this study represent regulatory associations derived from the integrated network structure, rather than direct experimental verification of specific modification events.

### 4.4. Parameter Estimation of GWGENs by Microarray Data and System Identification Method

We used the system identification method to identify the parameters of the PPIs Equation (1) using microarray data. It can be described by the linear regression form as follows:

(5) $$\begin{array}{l} p_{x}[n]=\left[p_{i}[n]p_{1}[n]\;\;p_{i}[n]p_{2}[n]\;\cdots \;p_{i}[n]p_{Yx}[n]\;1\right]\cdot \left[\begin{array}{l} a_{x1}\\ a_{x2}\\ \;\vdots \\ a_{xYx}\\ b_{x,PPIN} \end{array}\right]\\ \;+\;w_{x,PPIs}\left[n\right],\;\mathrm{for}\;x=1,\;\ldots ,\;X \end{array}$$

Equation (5) can be described as follows:

(6) $$p_{x}[n]=\Xi_{x,PPIs}^{T}[n]\cdot \theta_{x,PPIs}+w_{x,PPIs}\left[n\right],\;\mathrm{for}\;x=1,\;\ldots ,\;X$$

where $\Xi_{x,PPIs}^{T}[n]$ = $\left[p_{x}[n]p_{1}[n]\;\;p_{x}[n]p_{2}[n]\;\cdots \;p_{x}[n]p_{Yx}[n]\;1\right]$ represents the regression vector, based on the microarray data; $\theta_{x,PPIs}$ = $\left[\begin{array}{l} a_{x1}\\ a_{x2}\\ \;\vdots \\ a_{xYx}\\ b_{x,PPIN} \end{array}\right]$ represents the unknown parameter vector to be estimated for the *x*th protein in PPIs.

Equation (6) could be augmented to *N* samples of microarray data as follows:

(7) $$\left[\begin{array}{l} p_{x}[1]\\ \;\vdots \\ p_{x}[N] \end{array}\right]=\left[\begin{array}{l} \Xi_{x,PPIs}^{T}[1]\\ \;\vdots \\ \Xi_{x,PPIs}^{T}[N] \end{array}\right]\cdot \theta_{x,PPIs}+\left[\begin{array}{l} w_{x,PPIs}\left[1\right]\\ \;\vdots \\ w_{x,PPIs}\left[N\right] \end{array}\right]$$

Let Equation (7) be abbreviated as follows:

(8) $$P_{x}=\Omega_{x,PPIs}\cdot \theta_{x,PPIs}+\alpha_{x,PPIs}$$

In the next step, we apply the following least-squares estimation method to estimate the parameters $\theta_{x,PPIs}$:

(9) $${\hat{\theta }}_{x,PPIs}=\underset{\tau_{x,PPIs}}{\min}\frac{1}{2}\left\|\Omega_{x,PPIs}\cdot \right.\theta_{x,PPIs}-{\left.P_{x}\right\|}_{2}^{2}$$

After finishing the parameter interaction one protein by one protein, we could identify PPIs of PPIN in the GWGEN by the corresponding high-throughput data.

Furthermore, the inequality constraint on the least square parameter estimation in (S15) could guarantee miRNA non-post-transcriptional regulatory ability $-\sigma_{zr}$ must be non-positive.

Because candidate GWGEN is constructed by big database mining from all experimental and computational predictions which may contain some faulty information, for example, many false positives of protein interactions, transcriptional regulations and post-transcriptional regulatory abilities. Therefore, we use the system order detection method of AIC to prune the insignificant parameters in candidate GWGENs by deleting insignificant components out of the system order to obtain real GWGENs of three stages of asthma.

In the PPIs model (8), the AIC of the *x*th protein could be defined as follows:

(10) $$AIC_{x,PPIs}(Y_{x})=\log[\frac{1}{N}{\left(P_{x}-\Omega_{x,PPIs}\cdot {\hat{\theta }}_{x,PPIs}\right)}^{T}(P_{x}-\Omega_{x,PPIs}\cdot {\hat{\theta }}_{x,PPIs})]+\frac{2(Y_{x}+1)}{N}$$

where ${\hat{\theta }}_{x,PPIs}$ denotes the estimated parameters of protein *x* obtained from the solutions of the parameter estimation problem in (9). $\frac{1}{N}{\left(P_{x}-\Omega_{x,PPIs}\cdot {\hat{\theta }}_{x,PPIs}\right)}^{T}(P_{x}-\Omega_{x,PPIs}\cdot {\hat{\theta }}_{x,PPIs})$ indicates the estimated residual error. $Y_{x}$ in the second term of (25) denotes the system order. According to system identification theory [53], AIC is a tradeoff between the estimated residual error and system order, and will achieve the minimum at the real system order (i.e., the number of parameters). It can be realized that the minimum $AIC_{x,PPIs}(Y_{x})$ in (10) can be solved for the parameter number $Y_{x}^{*}$ of the real PPIs of protein x in the PPIs. The insignificant interactions out of $Y_{x}^{*}$ should be deleted as false positives from the PPIs of protein *x*. Then, by a similar procedure, one protein by one protein, we could obtain the real PPIs in GWGEN.

Using a similar system identification framework, the GRN, LRN, and MRN models were constructed. These models follow analogous regression formulations, data augmentation procedures, and constrained least-squares estimation schemes as described for the PPI model. For brevity, the detailed mathematical formulations and derivations are provided in the Supplementary Text S1.

By applying system identification and system order detection to the candidate GWGENs using stage-matched microarray data, real GWGENs corresponding to the quiet, exacerbation, and follow-up stages of asthma were inferred, as shown in Figure 2A–C. These real networks represent refined genetic and epigenetic regulatory architectures after removal of spurious interactions.

Comparison between the candidate and real GWGENs (Table 1) revealed substantial pruning of false-positive nodes across all three stages. Despite this reduction, the total number of retained interactions remained comparable among stages, indicating that core regulatory complexity was preserved following network refinement (Figure 2A–C).

To further examine shared and stage-specific components, a three-set Venn diagram of retained proteins was constructed (Figure 2D). Approximately 19.5–22.5% of proteins were unique to each stage, while a substantial fraction was shared across multiple stages. This result highlights the coexistence of a conserved core network and distinct stage-specific regulatory components, which likely contribute to differential pathogenic mechanisms during asthma progression.

The system parameters were estimated using a constrained least-squares estimation framework based on genome-wide microarray data from the GSE19301 dataset. Specifically, the regression formulations in (7), (S3), and (S8) were constructed for the PPIN, GRN, and LRN, respectively. The corresponding parameter vectors ($\hat{\theta }$), including interaction coefficients and basal activity terms, were obtained by solving the constrained optimization problems defined in (9), (S5), and (S10).

To determine the optimal system order and eliminate insignificant interactions, a stepwise selection procedure guided by the AIC was applied. The AIC balances model complexity and goodness of fit, and the optimal parameter set was selected by minimizing the AIC value. Interactions associated with non-optimal system orders were pruned as false positives, resulting in refined stage-specific GWGENs.

### 4.5. Extracting Core Network from the Real GWGEN by Using the PNP Method

It is essential to establish an integrated system network matrix *M* of a real GWGEN before we apply the PNP method to extract the core GWGENs from the real GWGENs. In addition, the system network matrix *M* involves the whole estimated system parameters in the real GWGENs as follows:

(11) $$M=\left[\begin{array}{c} \begin{array}{ccc} \begin{array}{ccc} {\hat{a}}_{11} & \cdots & {\hat{a}}_{1O}\\ \vdots & {\hat{a}}_{xo} & \vdots \\ {\hat{a}}_{X1} & \cdots & {\hat{a}}_{XO} \end{array} & \begin{array}{ccc} 0 & \cdots & 0\\ \vdots & 0 & \vdots \\ 0 & \cdots & 0 \end{array} & \begin{array}{ccc} 0 & \cdots & 0\\ \vdots & 0 & \vdots \\ 0 & \cdots & 0 \end{array}\\ \begin{array}{ccc} {\hat{c}}_{11} & \cdots & {\hat{c}}_{1O}\\ \vdots & {\hat{c}}_{io} & \vdots \\ {\hat{c}}_{I1} & \cdots & {\hat{c}}_{IO} \end{array} & \begin{array}{ccc} {\hat{d}}_{11} & \cdots & {\hat{d}}_{1U}\\ \vdots & {\hat{d}}_{iu} & \vdots \\ {\hat{d}}_{I1} & \cdots & {\hat{d}}_{IU} \end{array} & \begin{array}{ccc} {\hat{e}}_{11} & \cdots & {\hat{e}}_{1R}\\ \vdots & {\hat{e}}_{ir} & \vdots \\ {\hat{e}}_{I1} & \cdots & {\hat{e}}_{IR} \end{array}\\ \begin{array}{ccc} {\hat{S}}_{11} & \cdots & {\hat{S}}_{1O}\\ \vdots & {\hat{S}}_{ho} & \vdots \\ {\hat{S}}_{H1} & \cdots & {\hat{S}}_{HO} \end{array} & \begin{array}{ccc} {\hat{\Gamma }}_{11} & \cdots & {\hat{\Gamma }}_{1U}\\ \vdots & {\hat{\Gamma }}_{iu} & \vdots \\ {\hat{\Gamma }}_{H1} & \cdots & {\hat{\Gamma }}_{HU} \end{array} & \begin{array}{ccc} {\hat{\lambda }}_{11} & \cdots & {\hat{\lambda }}_{1R}\\ \vdots & {\hat{\lambda }}_{hr} & \vdots \\ {\hat{\lambda }}_{H1} & \cdots & {\hat{\lambda }}_{HR} \end{array}\\ \begin{array}{ccc} {\hat{\eta }}_{11} & \cdots & {\hat{\eta }}_{1O}\\ \vdots & {\hat{\eta }}_{zo} & \vdots \\ {\hat{\eta }}_{Z1} & \cdots & {\hat{\eta }}_{ZO} \end{array} & \begin{array}{ccc} {\hat{\delta }}_{11} & \cdots & {\hat{\delta }}_{1U}\\ \vdots & {\hat{\delta }}_{zu} & \vdots \\ {\hat{\delta }}_{Z1} & \cdots & {\hat{\delta }}_{ZU} \end{array} & \begin{array}{ccc} {\hat{\sigma }}_{11} & \cdots & {\hat{\sigma }}_{1R}\\ \vdots & {\hat{\sigma }}_{zr} & \vdots \\ {\hat{\sigma }}_{Z1} & \cdots \; & {\hat{\sigma }}_{ZR} \end{array} \end{array} \end{array}\right]\in {\mathbb{R}}^{\left(X+I+H+Z)\times (O+U+R\right)}$$

where ${\hat{a}}_{xo}$ were the corresponding components in ${\hat{\theta }}_{x,PPIs}$ by solving the parameter estimation problem in (9) and the system order detection problem in (10); where ${\hat{c}}_{io}$, ${\hat{d}}_{iu}$ and ${\hat{e}}_{ir}$ were the corresponding components in ${\hat{\theta }}_{i,GRN}$ by solving the parameter estimation problem in (S5) and the system order detection problem in (S16); where ${\hat{S}}_{ho}$, ${\hat{\Gamma }}_{iu}$ and ${\hat{\lambda }}_{hr}$ were the corresponding components in ${\hat{\theta }}_{h,LRN}$ by solving the parameter estimation problem in (S10) and the system order detection problem in (S17); where ${\hat{\eta }}_{zo}$, ${\hat{\delta }}_{zu}$ and ${\hat{\sigma }}_{zr}$ were the corresponding components in ${\hat{\theta }}_{z,MRN}$ by solving the parameter estimation problem in (S15) and the system order detection problem in (S18); the pruned false-positives in matrix *M* of GWGEN are padded with zeros. In general, a row vector in *M* represents the corresponding edges of a node in GWGEN.

In the network matrix *M*, the corresponding component is zero if a link does not appear in the candidate GWGEN or has been pruned via AIC. Then, we extract the core network of the real GWGEN by applying PNP to the network matrix *M*. PNP is realized on the basis of the singular value decomposition of *M* in the following:

(12) $$M=UDV^{T}$$

where *U* ∈ ${\mathbb{R}}^{\left(X+I+H+Z)\times (O+U+R\right)}$, *V* ∈ ${\mathbb{R}}^{\left(O+U+R)\times (O+U+R\right)}$, and D = diag ($d_{1}$, …, $d_{O+U+R}$) includes the *O* + *U* + *R* singular values of *M* in descending order, that is, $d_{1}$ ≥ … ≥ $d_{O+U+R}$. Notably, diag ($d_{1}$, $d_{2}$) indicates the diagonal matrix of $d_{1}$ and $d_{2}$. In addition, we can define the eigenexpression fraction ($E_{i}$) for the normalization of singular values as follows:

(13) $$E_{i}=\frac{d_{i}^{2}}{\sum_{i=1}^{O+U+R}d_{i}^{2}}$$

From the perspective of energy, the core network needs to maintain the system energy of the whole network structure. Thus, we choose the top *K* singular vectors of the network matrix *M* with the minimum *K* so that it leads to $\sum_{i=1}^{K}E_{i}$ ≥ 85% to represent at least 85% energy of the core network structure of a GWGEN, which is composed of these top *K* principal components. We retained the top principal components such that the cumulative explained variance exceeded 85% of the total energy. This threshold preserves the dominant regulatory patterns while effectively filtering out low-contribution or noisy interactions. The reduced matrix obtained from this projection defines the core GWGEN, highlighting key regulatory interactions underlying asthma progression.

Next, the projection of each row vector (the edges of nodes in GWGEN) of the network matrix *M* to these top *K* principal singular vectors of *V* is shown in the following,

(14) $$V(s_{\omega },i)=v_{s_{\omega },:}\cdot m_{:,i}^{T},\;\mathrm{for}\;s_{\omega }=1,\;\ldots ,\;X+I+H+Z\;i=1,\;\ldots ,\;K$$

where $v_{s_{\omega },:}$ represents the *s_ω_*-th row vector of *M*; $m_{:,i}$ represent the *i*th row vector of $V^{T}$. Finally, we apply the 2-norm projection value of each node, including gene, miRNA, lncRNA, protein, and protein complex in the real GWGEN, to the top *K* right singular vectors in the following:

(15) $$D(s_{\omega })={\left[\sum_{i=1}^{K}{\left[V(s_{\omega },i)\right]}^{2}\right]}^{\frac{1}{2}},\;\mathrm{for}s_{\omega }=1,\;\ldots ,\;X+I+H+Z,\;\mathrm{and}\;i=1,\;\ldots ,\;K$$

It is implied that if the projection value $D(s_{\omega })$ approaches zero, the contribution of the corresponding *s_ω_*-th node is negligible and largely independent of the core network structure formed by the top *K* singular vectors. Based on the projection values defined in Equation (15), we extract the core network by selecting proteins, genes, and miRNAs with the highest projection values. Importantly, this selection is further constrained by the regulatory connectivity inferred from the estimated optimal parameters ($\hat{\theta }$). Specifically, only nodes that participate in coherent regulatory paths, such as receptor-mediated signaling cascades involving proteins, TFs, and downstream target genes, are retained.

These regulatory paths are established from the parameter-estimated GWGEN, where edges represent statistically validated interactions after AIC-based pruning. Therefore, the resulting core network preserves both the dominant structural components identified by PNP and the biologically meaningful signal transduction cascades. The core GWGENs corresponding to the quiet, exacerbation, and follow-up stages of asthma are presented in Supplementary Figures S1–S3, respectively. This combined criterion avoids selecting isolated high-projection nodes that are not functionally connected within regulatory pathways.

### 4.6. Drug–Target Identification Using cMAP

To identify candidate drugs for reversing disease-associated gene expression patterns, we utilized the cMAP database [54], which provides gene expression profiles of human cells treated with a large number of small-molecule compounds.

The dataset used in this study contains expression responses of 14,207 genes to 1327 drugs across multiple human cell lines. To obtain a generalized drug effect, gene expression changes induced by each drug were averaged across all available cell lines.

For each asthma stage, target genes were first classified as upregulated or downregulated based on the inferred regulatory network analysis. Candidate drugs were then selected based on their ability to reverse these expression patterns, i.e., drugs that downregulate disease-upregulated genes and upregulate disease-downregulated genes.

## 5. Conclusions

In this study, we integrated medical big data from NCBI GEO microarray datasets with publicly available interaction databases to stratify asthma into three disease stages and reconstruct candidate genome-wide genetic and epigenetic networks. By employing a systems regression framework that incorporates protein–protein interactions, gene regulatory relationships, and lncRNA–miRNA regulation, we applied system identification and system order detection to eliminate false-positive interactions and infer real networks for each stage.

Because the inferred networks were too complex for direct interpretation, we applied PNP to extract core subnetworks and mapped these to KEGG pathways to elucidate stage-specific regulatory mechanisms. From the resulting core pathways, we identified key biomarkers that serve as potential drug targets.

The identified targets reflect the combined effects of microenvironmental perturbations, epigenetic modification, genetic variation, and post-transcriptional regulation. For the quiet stage, multi-molecule drug combinations were designed to modulate targets including *CYFIP2*, *IRF5*, *IRF1*, *HLA-G*, and *RHOB*, with the aim of improving cell adhesion, regulating inflammatory responses, enhancing innate immunity, and promoting tissue recovery. For the exacerbation stage, candidate targets such as *KLF4*, *ZEB1*, *IL4*, *IL10*, and *HOXA5* were selected to suppress epithelial–mesenchymal transition, reduce inflammation, and alleviate airway obstruction and hyperresponsiveness. For the follow-up stage, targets including *MUC2*, *NFKB2*, *SOCS3*, STAT1, and *SPDEF* were prioritized to mitigate airway remodeling, mucus hypersecretion, chronic inflammation, and steroid insensitivity.

By targeting regulatory mechanisms in a stage-dependent manner, the proposed systems biology framework provides a strategy for reducing asthma exacerbation frequency, improving clinical outcomes, and potentially lowering treatment costs.

Given the vast number of genes in the human genome, exhaustive enumeration of all possible gene–protein interactions is not feasible. Therefore, our candidate networks were restricted to interactions supported by existing literature and databases, ensuring interpretability of the inferred networks. Incorporating additional interactions, such as those inferred from cross-species homology, would substantially increase computational complexity and risk overfitting without experimental validation. Future studies may extend this framework by integrating comparative genomics across related species or disease models to uncover previously unrecognized regulatory mechanisms involved in asthma progression.

## Figures and Tables

**Table 1:** Number of nodes in candidate and real GWGENs across different asthma stages. Nodes include TFs, lncRNAs, miRNAs, receptors, and other proteins identified before and after network refinement for the quiet, exacerbation, and follow-up stages.

	Candidate	Quiet	Exacerbation	Follow-Up
TFs	1882	1664	1697	1698
lncRNAs	56	54	52	53
miRNAs	104	61	60	61
Receptors	1900	1751	1752	1750
Proteins	10,082	9901	9904	9906
Total nodes	14,024	13,431	13,465	13,468

**Table 2:** Number of edges in candidate and real GWGENs across different asthma stages. Despite substantial pruning of false-positive interactions during system identification, the overall network scale and interaction diversity were preserved across stages.

	Candidate	Quiet	Exacerbation	Follow-Up
T-G	62,864	56,734	56,539	56,531
T-T	25,395	19,400	20,048	20,472
T-R	13,992	12,446	12,642	12,634
T-L	101	84	82	85
T-M	88	83	82	83
L-G	25	20	17	17
L-T	48	20	36	40
M-G	7022	4345	4296	4403
M-T	1802	1181	1198	1193
M-R	1451	975	947	961
M-L	11	5	5	5
PPIs	2,785,732	707,264	707,324	640,352
Total edges	2,898,531	802,557	803,216	736,776

Abbreviations: T-G, transcriptional regulation of transcription factors (TFs) to genes; T-T, T-R, T-L, T-M, regulation of TFs to TFs, receptors, ligands, and miRNA-coding genes; L-G and L-T, ligand-mediated regulation to TF-coding genes and other genes; M-G, M-T, M-R, M-L, negative regulation of miRNAs to genes, TFs, receptors, and ligands; PPIs, protein–protein interactions in GWGENs.

**Table 3:** Enriched cellular functions and Kyoto Encyclopedia of Genes and Genomes (KEGG) pathways of stage-specific target genes identified by DAVID analysis. *p*-values were calculated using a modified Fisher’s exact test. No multiple-testing correction was applied, and pathways were ranked based on statistical significance and biological relevance.

	KEGG Pathway	Count	*p*-Value
Quiet	Regulation of actin cytoskeleton	196	2.1 × 10^−4^
	Cell adhesion molecules	122	5.8 × 10^−21^
	Toll-like receptor signaling pathway	98	2.4 × 10^−3^
	MAPK signaling pathway	247	1.1 × 10^−6^
	Pathways in cancer	312	3.0 × 10^−10^
Exacerbation	Cytokine-cytokine receptor interaction	239	3.1 × 10^−5^
	Chemokine signaling pathway	171	4.1 × 10^−4^
	Regulation of cytokine production	162	6.4 × 10^−8^
	Hematopoietic cell lineage	86	6.8 × 10^−7^
Follow-up	Jak–STAT signaling pathway	141	1.2 × 10^−3^
	Insulin signaling pathway	128	3.2 × 10^−5^
	Neurotrophin signaling pathway	118	4.4 × 10^−5^
	Apoptosis	83	7.5 × 10^−4^

**Table 4:** Network-guided integration of multi-molecule drug effects on stage-specific asthma biomarkers. The final result summarizes the overall regulatory tendency of the proposed multi-molecule drug combination at each disease stage, aiming to reverse disease-associated gene expression patterns rather than to quantify clinical efficacy.

Quiet	Drug Target	CYFIP2	HLA-G	IRF1	IRF5	RHOB
	Expression	+	+	−	−	+
	Drug effect					
	erythromycin	−	−	−	0	−
	ergocalciferol	0	−	−	+	−
	dexamethasone	+	−	+	+	+
	perhexiline	−	−	+		+
	MK-886	−	+	+	+	+
	Final result					
		−	−	+	+	+
Exacerbation	Drug target	IL4	KLF4	HOXA5	ZEB1	IL10
	Expression	+	+	+	+	−
	Drug effect					
	Trolox C	−	−	+	−	+
	budesonide	−	−	+	+	+
	tomatidine	−	−	−	−	−
	hesperetin	−	−	−	−	−
	trichostatin A	+	−	−	+	+
	Final result					
		−	−	−	−	+
Follow-up	Drug target	STAT1	NFKB2	MUC2	SPDEF	SOCS3
	Expression	+	+	+	+	−
	Drug effect					
	chloroquine	+	−	−	−	−
	imatinib	−	−	+	+	+
	carbamazepine	−	−	−	−	−
	iloprost	−	−	+	−	+
	luteolin	+	+	−	−	+
	Final result					
		−	−	−	−	+

“+” and “−” in the Expression rows indicate upregulation or downregulation of target gene expression inferred from the GSE19301 dataset. “+” and “−” in the Drug effect rows represent activation or inhibition of target gene expression inferred from cMap perturbation profiles, while “0” denotes no detectable effect.

## Data Availability

The datasets analyzed in this study are publicly available in the GEO repository under accession number GSE19301 (https://www.ncbi.nlm.nih.gov/geo/; accessed on 5 March 2023). Additional processed data and analysis results are available from the corresponding author upon reasonable request.

## Supplementary Materials

The following supporting information can be downloaded at: https://www.mdpi.com/article/10.3390/ijms27093708/s1.
