# Supplementary material for: Stage-Specific Reconstruction of Genome-Wide Genetic and Epigenetic Regulatory Networks Reveals Mechanistic Insights into Asthma Progression
Source: Int J Mol Sci. 2026 Apr 22;27(9):3708. doi: 10.3390/ijms27093708 (PMC13163776; doi:10.3390/ijms27093708)
Supplement: Supplementary file 1 [file ijms-27-03708-s001.zip › ijms-4254347-supplementary.pdf]

Supplementary Text S1. Detailed derivation of GRN, LRN, and MRN models

This section provides the complete mathematical formulations and parameter estimation procedures for the gene regulatory network (GRN), lncRNA regulatory network (LRN), and miRNA regulatory network (MRN) models.

These models follow the same system identification framework as the PPI model described in the main text, including regression formulation, data augmentation, constrained least-squares estimation, and AIC-based model order selection.

We used the system identification method to identify the accurate parameters of the gene regulatory eq. (2) as the following:

$$g_i[n]=\begin{bmatrix} p_1[n] & p_2[n] & \cdots & p_{O_i}[n] & l_1[n] & l_2[n] & \cdots & l_{U_i}[n] & g_i[n]k_1[n] & g_i[n]k_2[n] & \cdots & g_i[n]k_{R_i}[n] & 1 \end{bmatrix} \times \begin{bmatrix} c_{i1} \\ c_{i2} \\ \vdots \\ c_{O_i} \\ d_{i1} \\ d_{i2} \\ \vdots \\ d_{iUi} \\ -e_{i1} \\ -e_{i2} \\ \vdots \\ -e_{iRi} \\ b_{i,GRN} \end{bmatrix} + w_{i,GRN}[n], \text{for } i=1,...,I \quad \text{(S1)}$$

Eq. (S1) can be described as the following:

$$g_i[n]=\Xi^T_{i,GRN}[n]\cdot\theta_{x,GRN} + w_{i,GRN}[n], \text{ for } i=1,...,\mathrm{I} \quad (S2)$$

Eq. (S2) could be augmented to N samples of microarray data, which is indicated as the following:

$$\begin{bmatrix} g_i[1] \\ \vdots \\ g_i[N] \end{bmatrix} = \begin{bmatrix} \Xi^T_{i,GRN}[1] \\ \vdots \\ \Xi^T_{i,GRN}[N] \end{bmatrix} \cdot \theta_{i,GRN} + \begin{bmatrix} w_{i,GRN}[1] \\ \vdots \\ w_{i,GRN}[N] \end{bmatrix} \quad (S3)$$

Let eq. (S3) be abbreviated as follows:

$$G_i = \Omega_{i,GRN} \cdot \theta_{i,GRN} + \alpha_{i,GRN} \qquad (S4)$$

In the next step, we apply the following least-squares estimation method to estimate the parameters $\tau_{\mathrm{i,GRN}}$:

$$\hat{\theta}_{i,GRN} = \min_{\tau_{i,GRN}} \frac{1}{2} \left\| \Omega_{i,GRN} \cdot \theta_{i,GRN} - G_x \right\|_2^2 \qquad (S5)$$

$$\text{subject to} \begin{bmatrix} \overset{O_i+U_i}{\overleftrightarrow{0 \ \cdots \ 0}} & \overset{R_i}{\overleftrightarrow{1\,0\,0 \cdots 0\,0}} & 0 \\ 0 \ \cdots \ 0 & 0\,1\,0 \cdots 0\,0 & \vdots \\ \vdots \ \ddots \ \vdots & \vdots \ 0 \ \ddots \ \vdots \ \vdots & \vdots \\ 0 \ \cdots \ 0 & 0\,0 \ \cdots \ 1\,0 & 0 \\ 0 \ \cdots \ 0 & 0\,0 \ \cdots \ 0\,1 & 0 \end{bmatrix} \cdot \theta_{i,GRN} \le \begin{bmatrix} 0 \\ 0 \\ \vdots \\ 0 \\ 0 \end{bmatrix}$$

After finishing the parameter interaction one gene by one gene, we could identify GRN of GWGEN by the corresponding microarray data.

Furthermore, the inequality constraint on the least square parameter estimation in (14) could guarantees the miRNA non-post-transcriptional regulatory ability $-e_{ri}$ must be non-positive.

Using a similar approach, lncRNA regulatory network (LRN) model could be described below:

$$l_h[n] = \begin{bmatrix} p_1[n] & p_2[n] & \cdots & p_{Oh}[n] & l_1[n] & l_2[n] & \cdots & l_{U_h}[n] & l_h[n]k_1[n] & l_h[n]k_2[n] & \cdots & l_h[n]k_{R_h}[n] & 1 \end{bmatrix} \times \begin{bmatrix} S_{h1} \\ S_{h2} \\ \vdots \\ S_{hO_h} \\ \Gamma_{h1} \\ \Gamma_{h2} \\ \vdots \\ \Gamma_{hUh} \\ -\lambda_{h1} \\ -\lambda_{h2} \\ \vdots \\ -\lambda_{hRh} \\ \varphi_h \end{bmatrix} + w_{h,LRN}[n], \text{for } h = 1,...,H \qquad (S6)$$

The eq. (S6) can be described as the following:

$$l_h[n] = \Xi^T_{h,LRN}[n] \cdot \theta_{h,LRN} + w_{h,LRN}[n], \text{ for } h = 1,...,H \qquad (S7)$$

Eq. (S7) is augmented to N samples of microarray data, which is indicated as the following :

$$\begin{bmatrix} l_h[1] \\ \vdots \\ l_h[N] \end{bmatrix} = \begin{bmatrix} \Xi^T_{h,LRN}[1] \\ \vdots \\ \Xi^T_{h,LRN}[N] \end{bmatrix} \cdot \theta_{h,GRN} + \begin{bmatrix} w_{h,LRN}[1] \\ \vdots \\ w_{h,LRN}[N] \end{bmatrix} \qquad (S8)$$

Let eq. (S8) be abbreviated as follows:

$$L_h = \Omega_{h,LRN} \cdot \theta_{h,LRN} + \alpha_{h,LRN} \qquad (S9)$$

where $L_h = \begin{bmatrix} l_h[1] \\ \vdots \\ l_h[N] \end{bmatrix}$, $\Omega_{h,LRN} = \begin{bmatrix} \Xi^T_{h,LRN}[1] \\ \vdots \\ \Xi^T_{h,LRN}[N] \end{bmatrix}$, $\alpha_{h,LRN} = \begin{bmatrix} \Theta_h[1] \\ \vdots \\ \Theta_h[N] \end{bmatrix}$

In the next step, we apply the following least-squares estimation method to estimate the parameters $\theta_{\mathrm{h},LRN}$:

$$\hat{\theta}_{h,LRM} = \min_{\tau_{h,LRN}} \frac{1}{2} \left\| \Omega_{h,LRN} \cdot \theta_{h,LRN} - L_h \right\|_2^2 \qquad (S10)$$

$$\text{subject to } \begin{bmatrix} \overbrace{0 \;\cdots\; 0}^{O_h+U_h} & \overbrace{1\,0\,0\cdots 0\,0}^{R_h} & 0 \\ 0 \;\cdots\; 0 & 0\,1\,0\cdots 0\,0 & \vdots \\ \vdots \;\ddots\; \vdots & \vdots\; 0 \;\ddots\; \vdots\;\vdots & \vdots \\ 0 \;\cdots\; 0 & 0\,0\cdots 1\,0 & 0 \\ 0 \;\cdots\; 0 & 0\,0\cdots 0\,1 & 0 \end{bmatrix} \cdot \theta_{h,LRN} \le \begin{bmatrix} 0 \\ 0 \\ \vdots \\ 0 \\ 0 \end{bmatrix}$$

After finishing the parameter interaction one lncRNA by one lncRNA, we could identify LRN of GWGEN.

Furthermore, the inequality constraint on the least square parameter estimation in (S6) could guarantee miRNA non-post-transcriptional regulatory ability $-\lambda_{rh}$ must be non-positive.

Using a similar approach, miRNA regulatory network (MRN) model could be described below:

$$k_z[n] = \begin{bmatrix} p_1[n] & p_2[n] & \cdots & p_{Oz}[n] & l_1[n] & l_2[n] & \cdots & l_{U_z}[n] & k_z[n]k_1[n] & k_z[n]k_2[n] \end{bmatrix.$$

$$\left. \cdots \quad k_z[n]k_{R_z}[n] \quad 1 \right] \times \begin{bmatrix} \eta_{z1} \\ \eta_{z2} \\ \vdots \\ \eta_{zOz} \\ \delta_{z1} \\ \delta_{z2} \\ \vdots \\ \delta_{zUz} \\ -\sigma_{z1} \\ -\sigma_{z2} \\ \vdots \\ -\sigma_{zRz} \\ \Phi_z \end{bmatrix} + \zeta_z[n], for\ z = 1,...,Z \qquad (S11)$$

Eq. (S11) can be described as the following:

$$k_z[n] = \Xi^T_{z,MRN}[n] \cdot \theta_{z,MRN} + \zeta_z[n], \text{ for } z = 1,...,Z \qquad (S12)$$

Eq. (S12) is augmented to N samples of microarray data, which is indicated as follows:

$$\begin{bmatrix} k_z[1] \\ \vdots \\ k_z[N] \end{bmatrix} = \begin{bmatrix} \Xi^T_{z,MRN}[1] \\ \vdots \\ \Xi^T_{z,MRN}[N] \end{bmatrix} \cdot \theta_{z,GRN} + \begin{bmatrix} \zeta_z[n] \\ \vdots \\ \zeta_z[N] \end{bmatrix} \qquad (S13)$$

Let eq. (S13) is abbreviated as follows:

$$K_z = \Omega_{z,MRN} \cdot \theta_{z,MRN} + \alpha_{z,MRN} \qquad (S14)$$

In the next step, we apply the following least-squares estimation method to estimate the parameters $\theta_{\text{z,MRN}}$:

$$\hat{\theta}_{\text{z,MRN}} = \min_{\tau_{\text{z,MRN}}} \frac{1}{2} \left\| \Omega_{\text{z,MRN}} \cdot \theta_{\text{z,MRN}} - K_z \right\|_2^2 \qquad (S15)$$

$$\text{subject to} \begin{bmatrix} \overset{O_z+U_z}{\overleftrightarrow{0 \ \cdots \ 0}} & \overset{R_z}{\overleftrightarrow{1\,0\,0 \cdots 0\,0}} & 0 \\ 0 \ \cdots \ 0 & 0\,1\,0 \cdots 0\,0 & \vdots \\ \vdots \ \ddots \ \vdots & \vdots \ 0 \ \ddots \ \vdots \ \vdots & \vdots \\ 0 \ \cdots \ 0 & 0\,0\,0 \cdots 1\,0 & 0 \\ 0 \ \cdots \ 0 & 0\,0\,0 \cdots 0\,1 & 0 \end{bmatrix} \cdot \theta_{\text{z,MRN}} \le \begin{bmatrix} 0 \\ 0 \\ \vdots \\ 0 \\ 0 \end{bmatrix}$$

After finishing this parameter interaction one miRNA by one miRNA, we could identify MRN of GWGEN.

Similarly, in GRN model in (13), AIC of the *i*th gene could be defined as follows:

$$AIC_{i,GRN}(O_i, U_i, R_i) = \log[\frac{1}{N}(G_i - \Omega_{i,GRN} \cdot \hat{\theta}_{i,GRN})^T (G_i - \Omega_{i,GRN} \cdot \hat{\theta}_{i,GRN})] + \frac{2(O_i + U_i + R_i + 1)}{N} \qquad (S16)$$

where $\hat{\theta}_{\text{i,GRN}}$ denotes the estimated parameters of genes by the least square parameter estimation in (S5), and $\frac{1}{N}(G_i - \Omega_{i,GRN} \cdot \hat{\theta}_{i,GRN})^T (G_i - \Omega_{i,GRN} \cdot \hat{\theta}_{i,GRN})$ indicates the estimated residual error. It can be realized that the minimum $AIC_{i,GRN}(O_i, U_i, R_i)$ in (S16) can be achieved at the parameter number O^*_i, U^*_i and R^*_i of the real gene/miRNA/ lncRNA regulations of gene in GRN. Therefore, the insignificant regulations out of real regulation orders O^*_i, U^*_i and R^*_i should be pruned away one gene by one gene to obtain the real GRN in GWGEN.

Similarly, in LRM model in (S10), AIC of the *h*th lncRNA could be defined as follows:

$$AIC_{h,LRN}(O_h,U_h,R_h)=\log[\frac{1}{N}(L_h - \Omega_{h,LRN}\times\hat{\theta}_{h,})^T(L_h - \Omega_{h,LRN}\times\hat{\theta}_{h,LRN})] \quad (S17)$$
$$+\frac{2(O_i+U_i+R_i+1)}{N}$$

where $\hat{\theta}_{\mathrm{h,LRN}}$ denote the estimated parameters of the *h*th lncRNA from the least square parameter estimation in (S11), and $\frac{1}{N}(L_h-\Omega_{h,LRN}\cdot\hat{\theta}_{h,LRN})^T(L_h-\Omega_{h,LRN}\cdot\hat{\theta}_{h,LRN})$ indicates the estimated residual error. It can be realized that the minimum $AIC_{h,LRN}(O_h,U_h,R_h)$ in (S17) can be achieved at the number O^*_i, U^*_i and R^*_i of the real gene/miRNA/lncRNA regulations of lncRNA in LRM. Therefore, the insignificant regulations out of real regulation orders O^*_i, U^*_i and R^*_i should be pruned away one lncRNA by one lncRNA to obtain the real LRM in GWGEN.

Similarly, in MRN in (S14), AIC of the *z*th could be defined as follows:

$$AIC_{z,MRN}(O_z,U_z,R_z)=\log[\frac{1}{N}(K_z-\Omega_{z,MRN}\cdot\hat{\theta}_{z,MRN})^T(K_z-\Omega_{z,MRN}\cdot\hat{\theta}_{z,MRN})] \quad (S18)$$
$$+\frac{2(O_z+U_z+R_z+1)}{N}$$

where $\hat{\theta}_{\mathrm{z,MRN}}$ denotes the estimated parameters of genes obtained from the least square the parameter estimation in (S15), and $\frac{1}{N}(K_z-\Omega_{z,MRN}\cdot\hat{\theta}_{z,MRN})^T(K_z-\Omega_{z,MRN}\cdot\hat{\theta}_{z,MRN})$ indicates the estimated residual error. It can be realized that the minimum $AIC_{z,MRN}(O_z,U_z,R_z)$ in (S18) can be achieved at the number O^*_i, U^*_i and R^*_i of the real gene/miRNA/lncRNA regulations of miRNA in MRN. Therefore, the insignificant regulations out of real regulation orders O^*_i, U^*_i and R^*_i should be pruned away one miRNA by one miRNA to obtain the real MRN in GWGEN.

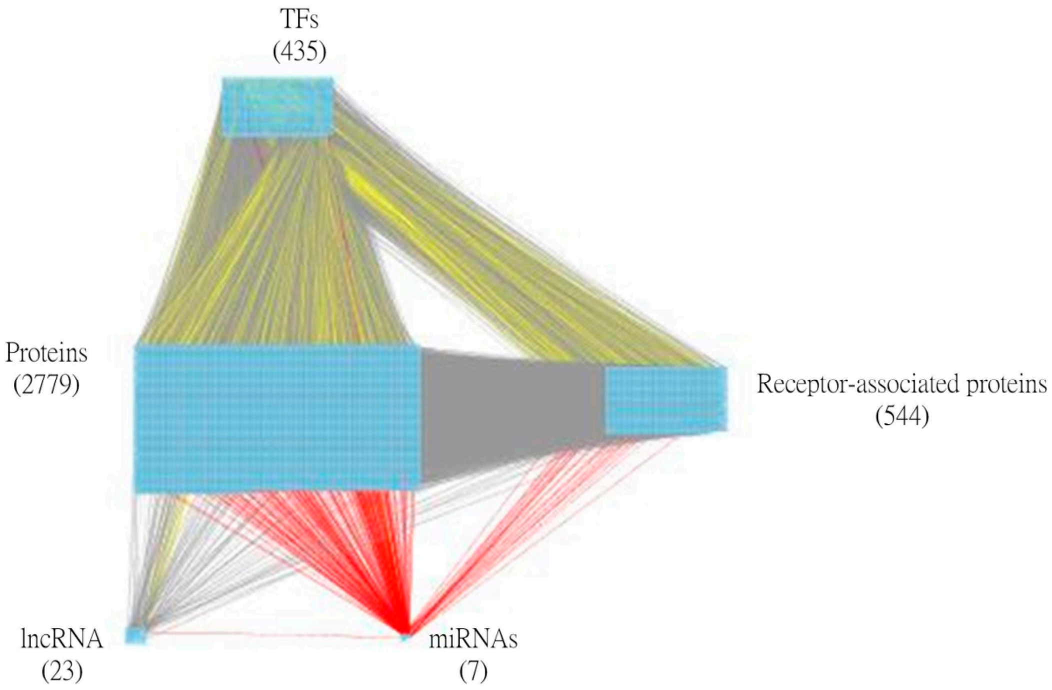

Supplemental Figure 1. The core GWGEN of quiet stage asthma.

This figure indicates the identified core GWGEN of quiet stage asthma. The grey lines represent protein-protein interactions, the yellow lines indicate the transcriptional regulations; the red lines denote the miRNA post-transcriptional regulations and the square blocks represent receptors, proteins/genes, lncRNAs, TFs and miRNAs, respectively.

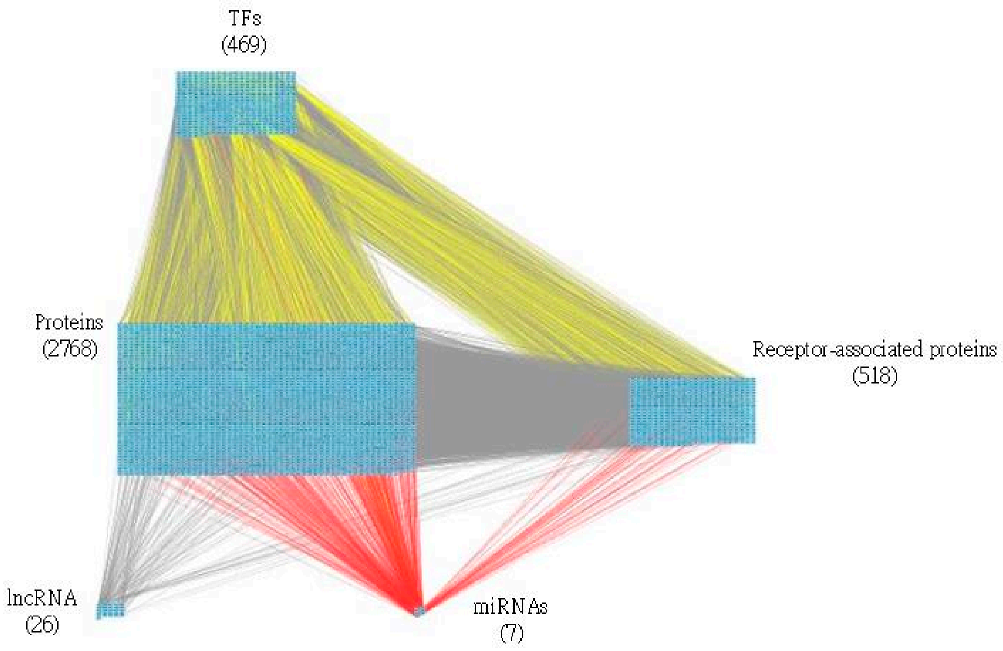

Supplemental Figure 2. The core GWGEN of exacerbation stage asthma.

This figure indicates the identified core GWGEN of exacerbation stage asthma. The grey lines represent protein-protein interactions, the yellow lines indicate the transcriptional regulations; the red lines denote the miRNA post-transcriptional regulations and the square blocks represent receptors, proteins/genes, lncRNAs, TFs and miRNAs, respectively.

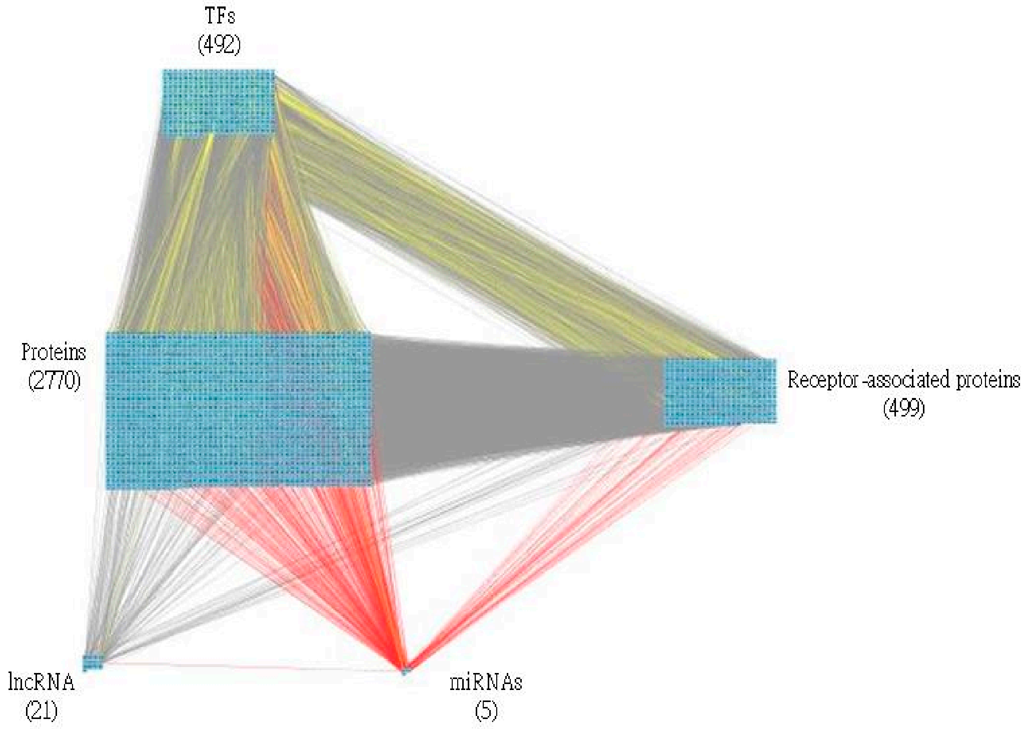

Supplemental Figure 3. The core GWGEN of follow-up stage asthma

This figure indicates the identified core GWGEN of follow-up stage asthma. The grey lines represent protein-protein interactions, the yellow lines indicate the transcriptional regulations; the red lines denote the miRNA post-transcriptional regulations and the square blocks represent receptors, proteins/genes, lncRNAs, TFs and miRNAs, respectively.
